# N4‐Acetylcytidine‐Mediated CD2BP2‐DT Drives YBX1 Phase Separation to Stabilize CDK1 and Promote Breast Cancer Progression

**DOI:** 10.1002/advs.202411834

**Published:** 2025-02-20

**Authors:** Hongyu Wang, Bozhi Zhao, Jiayu Zhang, Qunyu Hu, Linlin Zhou, Yinghui Zhang, Yixin Cai, Yuansong Qu, Tao Jiang, Dongwei Zhang

**Affiliations:** ^1^ Department of General Surgery The Second Affiliated Hospital of Harbin Medical University Harbin 150086 China; ^2^ Department of General Surgery The Affiliated Hospital of Xuzhou Medical University Institute of Digestive Diseases Xuzhou Medical University Xuzhou 221002 China

**Keywords:** breast cancer, CD2BP2‐DT, N4‐acetylcytidine, phase separation, proliferation, YBX1

## Abstract

Long noncoding RNAs (lncRNAs) play critical roles in the initiation and progression of breast cancer. However, the specific mechanisms and biological functions of lncRNAs in breast cancer remain incompletely understood. Bioinformatics analysis identifies a novel lncRNA, CD2BP2‐DT, that is overexpressed in breast cancer and correlates with adverse clinicopathological features and poor overall survival. Both in vivo and in vitro experiments demonstrate that CD2BP2‐DT promotes proliferation of breast cancer cells. Mechanistically, NAT10 mediates the N4‐acetylcytidine (ac4C) modification of CD2BP2‐DT, enhancing its RNA stability and expression. More importantly, CD2BP2‐DT enhances the stability of CDK1 mRNA by mediating YBX1 phase separation, thereby promoting the proliferation of breast cancer cells. In conclusion, the lncRNA CD2BP2‐DT is identified as a crucial driver of breast cancer cell proliferation through the YBX1/CDK1 axis, highlighting its potential as a promising biomarker and therapeutic target for breast cancer.

## Graphical abstract

The long non‐coding RNA (lncRNA) CD2BP2‐DT is upregulated in breast cancer and correlates with poor patient prognosis. Mechanistically, CD2BP2‐DT drives YBX1 phase separation to stabilize CDK1 and promote breast cancer progression. Moreover, NAT10‐mediated N4‐acetylcytosine (ac4C) modification further enhances CD2BP2‐DT expression. These findings highlight CD2BP2‐DT as a promising therapeutic target for breast cancer.

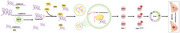

## 1 Introduction

The International Agency for Research on Cancer indicates that breast cancer is the most common malignancy and the leading cause of cancer‐related death among women.^[^ ^1^ ^]^ Despite significant advancements in surgical resection, chemotherapy, radiotherapy, endocrine therapy, and molecular targeted therapy, considerable challenges persist in the management of breast cancer.^[^ ^2^, ^3^ ^]^ Therefore, novel molecular markers and therapeutic targets are essential for enhancing treatment outcomes in breast cancer patients.

Long noncoding RNAs (lncRNAs) have been reported to play critical roles in mediating key biological processes, including cell differentiation, proliferation, and immune response, by reshaping chromatin and genome structure, regulating transcription, and modulating mRNA stability, translation, and post‐translational modification.^[^ ^4^, ^5^, ^6^, ^7^, ^8^ ^]^ An accumulating body of research indicates that the dysregulation of lncRNAs is pivotal in the initiation and progression of breast cancer.^[^ ^9^ ^]^ For example, lncRNA PRBC has been demonstrated to induce autophagy and promote breast cancer progression by modulating PABPC1‐mediated mRNA stabilization.^[^ ^10^ ^]^ Conversely, lncRNA‐BC069792 inhibits breast cancer progression by targeting KCNQ4.^[^ ^11^ ^]^ However, numerous lncRNAs with significant functions remain uncharacterized. Investigating and identifying these novel key genes are crucial for providing new molecular markers and therapeutic targets for patients with breast cancer.

Liquid‐liquid phase separation (LLPS) is a biological phenomenon in which macromolecules form distinct biomolecular condensates through various interactions. This process is essential for the concentration and functional regulation of biological macromolecules, including proteins and RNA.^[^ ^12^, ^13^ ^]^ LLPS has been widely observed to regulate critical cellular processes in cancer cells through the formation of biomolecular condensates, which fundamentally disrupt cellular homeostasis and promote tumorigenesis.^[^ ^14^, ^15^ ^]^ For instance, DAZAP1 promotes COX16 expression via LLPS, thereby driving tumor growth and metastasis.^[^ ^16^ ^]^ However, the precise connection between molecular condensates and cancer cell pathophysiology remains elusive.

Most RNA‐binding proteins (RBPs) are predisposed to phase separation due to the presence of intrinsically disordered regions (IDRs).^[^ ^17^ ^]^ RNAs, particularly lncRNAs, specifically bind to RBPs due to their extensive length and unique secondary structures, thereby enhancing LLPS.^[^ ^18^, ^19^ ^]^ Several lncRNAs have been identified as pivotal mediators of LLPS involving RBPs in cancer cells. Zhu et al. reported that the lncRNA MNX1‐AS1 promotes IGF2BP1 phase separation in lung cancer, thereby driving c‐Myc‐mediated cell cycle progression and proliferation.^[^ ^20^ ^]^ Additionally, Liu et al. demonstrated that the lncRNA MALR facilitates ILF3 LLPS to promote HIF1α signaling in esophageal cancer.^[^ ^21^ ^]^ However, the additional crosstalk between lncRNAs and LLPS of RBPs remains to be fully characterized. Therefore, further exploration of these interactions from a phase separation perspective may provide new insights into tumor pathology and open new avenues for disease investigation and treatment.

In this study, we identified a novel lncRNA, CD2BP2‐DT, which is significantly upregulated in breast cancer cell lines and tissues. The upregulation of CD2BP2‐DT is positively correlated with tumor size and poor overall survival in breast cancer patients. Additionally, CD2BP2‐DT was found to promote proliferation of breast cancer cells both in vitro and in vivo. Mechanistically, CD2BP2‐DT enhances the stabilization of CDK1 mRNA by mediating the LLPS of YBX1. Moreover, NAT10 mediated the N4‐acetylcytidine (ac4C) modification maintains the stability of CD2BP2‐DT and increases its expression. These findings establish CD2BP2‐DT as a crucial gene and highlight its potential as a promising therapeutic target for breast cancer.

## 2 Result

### 2.1 Identification of CD2BP2‐DT and its Clinical Significance in Breast Cancer

**Figure 1:**
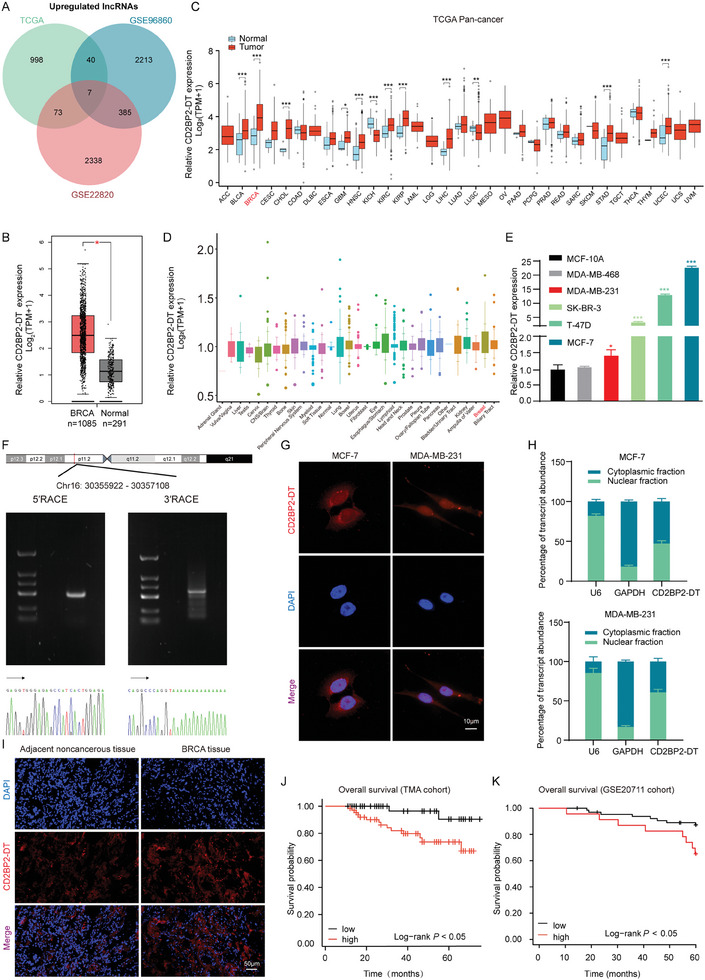
Identification of CD2BP2‐DT and analysis of its clinical significance in breast cancer. A) Venn diagram showing overlapping upregulated lncRNAs in GEO and TCGA datasets. B) The CD2BP2‐DT expression between breast cancer tissues and normal samples in the TCGA and GTEx databases (BRCA: breast cancer. BRCA, n = 1085; Normal, n = 291). C) Comparison of CD2BP2‐DT expression between different cancers and corresponding normal tissues in the TCGA database. D) The CD2BP2‐DT expression in cell lines was characterized using the CCLE database. E) Relative expression of CD2BP2‐DT in a normal mammary epithelial cell line (MCF‐10A) and breast cancer cell lines (MDA‐MB‐468, SK‐BR‐3, MDA‐MB‐231, T‐47D, and MCF‐7) (n = 3). F) CD2BP2‐DT sequence was confirmed by 5′ and 3′ cDNA ends assays. G) RNA‐FISH images showing the localization of CD2BP2‐DT in breast cancer cells. CD2BP2‐DT probes are labeled in red, and nuclei are counterstained with DAPI (scale bars, 10 µm). H) Cytoplasmic and nuclear fractions were extracted from breast cancer cells and analyzed for CD2BP2‐DT expression using qRT‐PCR. U6 served as the nuclear marker, while GAPDH was used as a cytoplasmic marker. I) Representative images illustrating CD2BP2‐DT expression in breast cancer TMA as detected by FISH staining (Adjacent noncancerous tissue, n = 114; BRCA, n = 114; scale bars, 50 µm). J) Kaplan‐Meier survival curve analysis of overall survival rates in breast cancer patients based on CD2BP2‐DT expression in the TMA cohort (n = 114). K) Overall survival curves for CD2BP2‐DT were analyzed using the GSE20711 dataset. (n = 88). The data in (E) were analyzed using one‐way ANOVA test, while survival comparisons in (J) and (K) were performed using the Log‐rank test. Results are presented as mean ± SD. Significance levels are indicated as *p < 0.05 and ***p < 0.001.

To identify lncRNAs that are highly expressed in breast cancer, we retrieved the GSE22820 dataset, which contains 176 breast cancer samples and 10 normal breast samples, as well as the GSE96860 dataset, which includes five molecular subtypes of breast cancer and two normal breast cell lines, from the Gene Expression Omnibus (GEO) database. Additionally, we obtained transcriptome data from The Cancer Genome Atlas (TCGA) database. Differentially expressed genes (DEGs) were identified using thresholds of |log FC| ≥ 1 and an adjusted *p* value < 0.05 (Figure S1A–C, Supporting Information). A Venn diagram showed that seven lncRNAs were upregulated in both breast cancer tissues and cell lines (**Figure** **1**A). Analysis of the GEPIA 2 platform indicated that CD2BP2‐DT was the only candidate with a statistically significant upregulation (Figure 1B; Figure S1D, Supporting Information). Given that CD2BP2‐DT is a novel lncRNA that has not been well characterized, we further explored its expression across different cancers. Pan‐cancer analysis demonstrated that CD2BP2‐DT was highly expressed in breast cancer, bladder urothelial carcinoma, and several other solid tumors compared to their corresponding normal tissues (Figure 1C). Furthermore, an analysis of data from the Cancer Cell Line Encyclopedia (CCLE) database indicated that the expression levels of CD2BP2‐DT were significantly elevated in breast cancer cell lines compared to most cell lines derived from other tissue types (Figure 1D). Validation in cell lines revealed that CD2BP2‐DT was significantly upregulated in MCF‐7, T‐47D, SK‐BR‐3, and MDA‐MB‐231 cells compared to the MCF‐10A cell line (Figure 1E). CD2BP2‐DT, located on chromosome 16 (30355922‐30357108), was characterized by rapid amplification of the 5′ and 3′ cDNA ends assays. Sanger sequencing was then performed to precisely determine the full‐length sequence of CD2BP2‐DT. The results indicated that CD2BP2‐DT is a 1215‐nt lncRNA (Figure 1F). Recent studies have demonstrated that some lncRNAs function through encoding tumor‐related peptides.^[^ ^22^ ^]^ The coding potential of CD2BP2‐DT was evaluated using the Coding Potential Calculator 2 (CPC2) and the Coding Potential Assessment Tool (CPAT), with ACTIN and GAPDH utilized as positive controls. Both CPC2 and CPAT indicated that CD2BP2‐DT has weak protein‐coding potential (Figure S1E, Supporting Information). Subcellular localization analysis using the lncATLAS database revealed that CD2BP2‐DT is localized in both the cytoplasm and nucleus of tumor cells (Figure S1F, Supporting Information). RNA fluorescence in situ hybridization (FISH) showed that CD2BP2‐DT is localized in both the cytoplasm and nucleus of breast cancer cells (Figure 1G; Figure S1G, Supporting Information), which was further supported by nucleocytoplasmic separation analyses (Figure 1H; Figure S1H, Supporting Information).

Additionally, FISH assays conducted on a tissue microarray (TMA) containing 114 paired breast cancer and adjacent normal tissue samples (ANTs) identified significant upregulation of CD2BP2‐DT in breast cancer tissues (Figure 1I). To evaluate the clinical significance of CD2BP2‐DT in this cohort, a chi‐square analysis was utilized to examine the correlation between CD2BP2‐DT expression and clinicopathological characteristics. Notably, the expression of CD2BP2‐DT was positively correlated with tumor size (*P* = 0.012) (Table S1, Supporting Information). Furthermore, Kaplan‐Meier survival analysis showed that high levels of CD2BP2‐DT were significantly associated with poor overall survival (Figure 1J). This finding was independently validated using the GSE20711 dataset (Figure 1K).

### 2.2 CD2BP2‐DT Promotes the Proliferation of Breast Cancer Cells In Vivo and In Vitro

**Figure 2:**
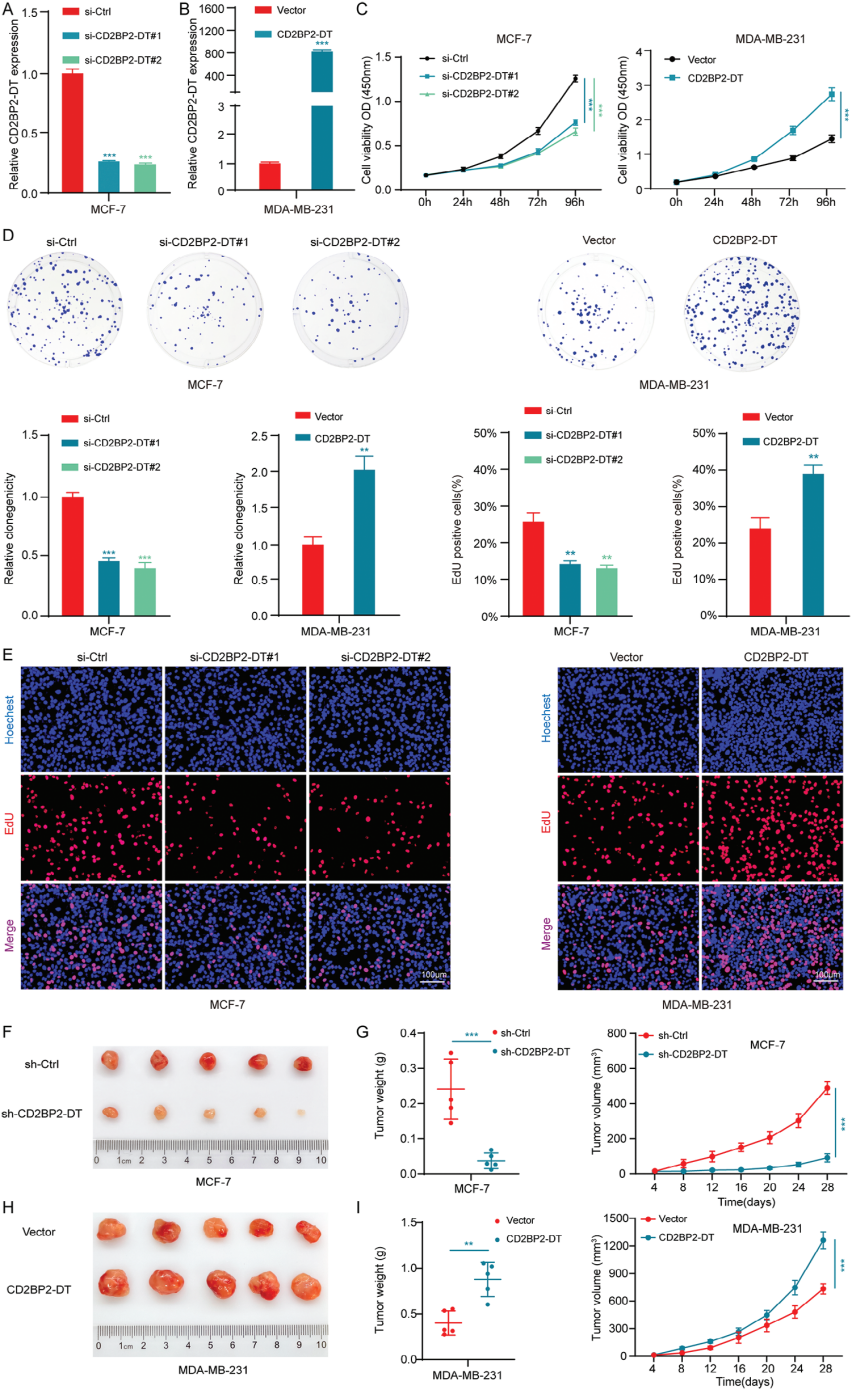
CD2BP2‐DT promotes the growth of breast cancer cells in vitro and in vivo. A) Assessment of the knockdown efficiency of CD2BP2‐DT siRNA using qRT‐PCR (n = 3). B) Evaluation of the overexpression efficiency of CD2BP2‐DT via qRT‐PCR (n = 3). C) The viability of breast cancer cells with CD2BP2‐DT knockdown or overexpression was assessed using CCK‐8 assays at the specified time points (n = 3). D,E) Colony formation (D) assays and EdU (E) assays were conducted to evaluate the proliferation capacity of breast cancer cells following CD2BP2‐DT overexpression or knockdown (n = 3; scale bars, 100 µm). F,H) A subcutaneous xenograft tumor experiment in nude mice was conducted to evaluate the effect of CD2BP2‐DT on the proliferation of breast cancer cells. G,I) Growth volume curve and tumor mass analysis of subcutaneous xenograft tumors (n = 5). Data in (A), (D), and (E) were analyzed using one‐way ANOVA test, Data in (B), (D), (E), (G), and (I) were calculated by unpaired Student's *t*‐test. Data in (C), (G), and (I) were calculated by two‐way ANOVA test. Results are presented as mean ± SD. Significance levels are indicated as **p < 0.01 and ***p < 0.001.

To explore the biological role of CD2BP2‐DT in breast cancer cells, we designed two siRNAs (si‐CD2BP2‐DT#1 and si‐CD2BP2‐DT#2), one shRNA (sh‐CD2BP2‐DT) targeting CD2BP2‐DT, and an overexpression plasmid. MCF‐7 and T‐47D cell lines, which exhibit the highest levels of CD2BP2‐DT expression, were selected for knockdown experiments. In contrast, MDA‐MB‐231 cells were chosen for overexpression experiments. The efficiency of knockdown and overexpression was confirmed by qRT‐PCR assays (**Figure** **2**A and B; Figure S2A, Supporting Information). The Cell Counting Kit‐8 (CCK‐8), 5‐Ethynyl‐2′‐deoxyuridine (EdU) and colony formation assays demonstrated that knockdown of CD2BP2‐DT significantly inhibited breast cancer cell proliferation, while overexpression of CD2BP2‐DT promoted proliferation (Figure 2C–E; Figure S2B–D, Supporting Information). To further investigate the effect of CD2BP2‐DT on breast cancer cell proliferation in vivo, xenograft tumor models were established in nude mice. The results showed that CD2BP2‐DT downregulation significantly inhibited xenograft tumor proliferation in vivo, while CD2BP2‐DT overexpression significantly promoted proliferation (Figure 2F–I). In addition, we investigated whether CD2BP2‐DT promotes the stemness of breast cancer cells. Immunohistochemical (IHC) staining showed no significant differences in the expression of CD133, CD44, and SOX2 in xenograft tumors (Figure S2E, Supporting Information). These findings demonstrate that CD2BP2‐DT promotes proliferation but has no effect on stemness of breast cancer.

### 2.3 CD2BP2‐DT Interacts with YBX1 to Promote Breast Cancer Cell Proliferation

**Figure 3:**
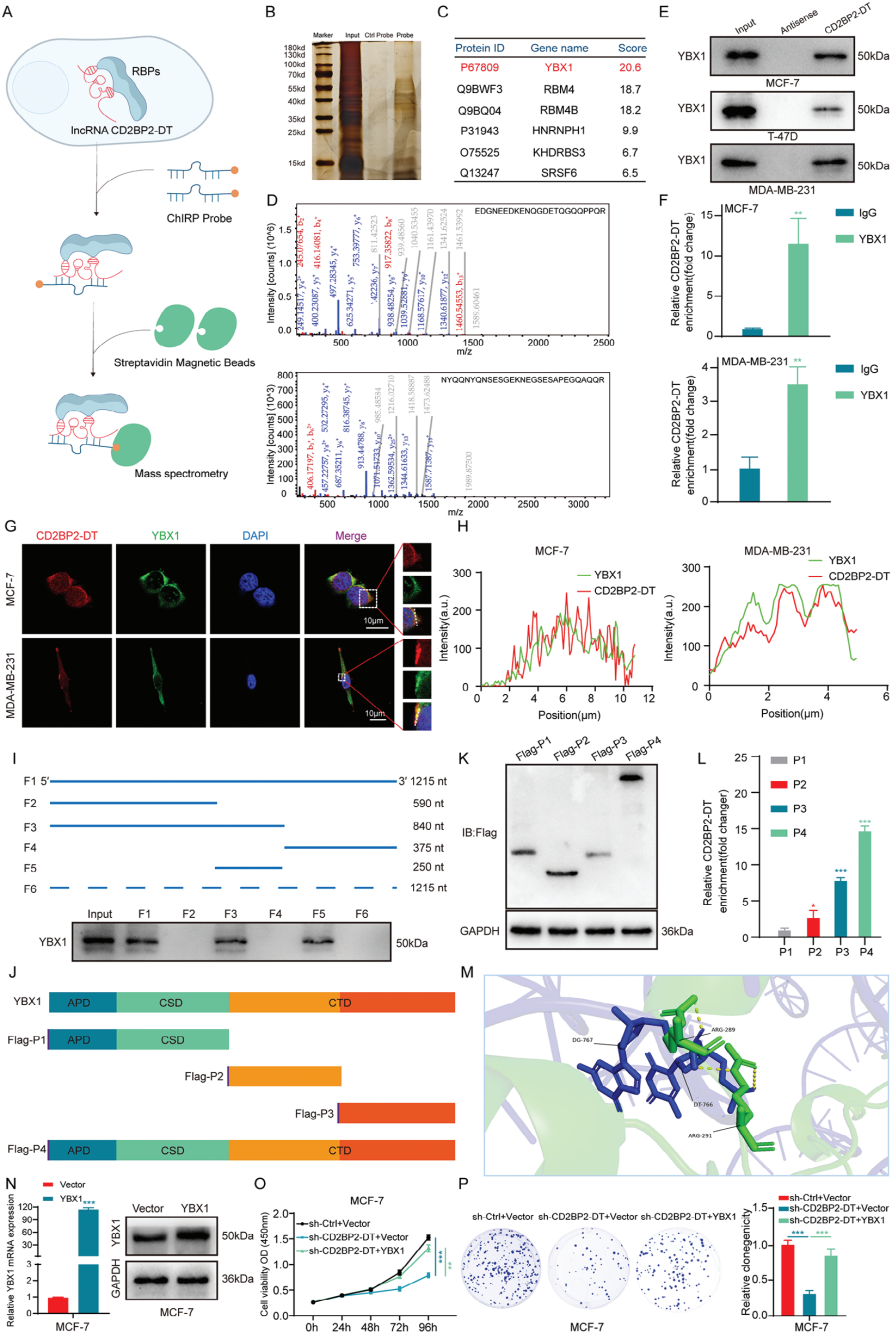
CD2BP2‐DT interacts with YBX1 to promote breast cancer cell proliferation. A) Schematic workflow of the ChIRP assay and MS used to identify CD2BP2‐DT binding proteins. B) Silver‐stained images of proteins pulled down by the CD2BP2‐DT probe and control probe in ChIRP analysis. C) Overlap analysis was performed between MS detection (35–55 kDa) and catRAPID prediction (Z‐score > 0.5), with results ranked according to MS scores. D) Ion chromatogram of a representative YBX1 peptide interacting with CD2BP2‐DT, as identified by mass spectrometry. E) Biotin‐labeled CD2BP2‐DT RNA pull‐down assay followed by Western blot analysis. F) The enrichment of CD2BP2‐DT by anti‐YBX1 in breast cancer cells was demonstrated through qRT‐PCR analysis (n = 3). G) RNA FISH and immunofluorescence experiments were conducted to analyze the co‐localization of CD2BP2‐DT with YBX1 (scale bars, 10 µm). H) Fluorescence co‐localization was statistically analyzed using image J software. I) Schematic representation of truncated CD2BP2‐DT fragments and Western blotting analysis of YBX1 in samples pulled down by biotin‐labeled CD2BP2‐DT. J) Schematic diagram of Flag‐tagged full‐length YBX1 and truncated YBX1 constructs. K) Western blotting analysis of breast cancer cells transfected with Flag‐tagged YBX1 constructs. L) RIP assay conducted in breast cancer cells utilizing Flag antibody, followed by the quantification of CD2BP2‐DT enrichment through qRT‐PCR (n = 3). M) The binding of CD2BP2‐DT and YBX1 was simulated by molecular docking. N) Western blotting and qRT‐PCR were employed to confirm the overexpression efficiency of YBX1 in breast cancer cells (n = 3). O,P) CCK‐8 (O) and colony formation (P) assays were used to analyze the effects of YBX1 overexpression on CD2BP2‐DT‐mediated breast cancer cell proliferation (n = 3). Data in (F), (L), and (N) were calculated by unpaired Student's *t*‐test. Data in (P) were analyzed using one‐way ANOVA test. Data in (O) were calculated by two‐way ANOVA test. Results are presented as mean ± SD. Significance levels are indicated as *p < 0.05, **p < 0.01, and ***p < 0.001.

Accumulating studies have shown that lncRNA‐RBP interactions play a crucial role in cancer progression. Non‐coding lncRNAs primarily function by establishing interactions with proteins and nucleic acids.^[^ ^23^ ^]^ To identify proteins that may interact with CD2BP2‐DT, we performed Chromatin Isolation by RNA Purification (ChIRP) experiments followed by mass spectrometry (MS) analysis (**Figure** **3**A). Silver staining of eluted proteins from the ChIRP experiment revealed distinct bands between 35 and 55 kDa in the CD2BP2‐DT probe pull‐down group that were absent in the control group (Figure 3B), suggesting specific protein interactions with CD2BP2‐DT. To further characterize potential binding partners, an overlap analysis was performed between candidate proteins identified in the 35 to 55 kDa range by MS analysis and those predicted to bind to CD2BP2‐DT using the catRAPID online tool. The results showed that YBX1 exhibited the highest score in the MS analysis (Figure 3C) and was selected as the binding partner of CD2BP2‐DT. In addition, we extracted the ion chromatograms and amino acid sequences of the YBX1 peptides from the MS data (Figure 3D) which represents potential key sites for the interaction between YBX1 and CD2BP2‐DT. YBX1 belongs to the RBP family and is a multifunctional oncoprotein involved in various biological processes, including DNA repair, pre‐mRNA splicing, mRNA transcription, stability, and regulation of translation.^[^ ^24^ ^]^ It is widely implicated in cancer cell proliferation, aging, and drug resistance processes.^[^ ^25^ ^]^ Recent studies have shown that YBX1 is significantly overexpressed in breast cancer and plays a crucial role in promoting tumor progression.^[^ ^26^, ^27^, ^28^ ^]^ Kaplan‐Meier survival analysis revealed that elevated YBX1 expression is associated with poor prognosis in breast cancer patients. (Figure S3A, Supporting Information). To confirm the interaction between CD2BP2‐DT and YBX1 in breast cancer cells, we conducted biotin‐labeled RNA pull‐down and RNA immunoprecipitation (RIP) assays (Figure 3E,F). Additionally, colocalization analyses using FISH and immunofluorescence (IF) revealed that CD2BP2‐DT and YBX1 are predominantly co‐localized in the cytoplasm of breast cancer cells (Figure 3G; Figure S3B, Supporting Information). The fluorescence co‐localization was statistically analyzed using Image J software (Figure 3H; Figure S3C, Supporting Information). To further explore the specific binding site of CD2BP2‐DT with YBX1, we utilized RNAfold software to predict the secondary structure of CD2BP2‐DT (Figure S3D, Supporting Information) and created a biotinylated full‐length CD2BP2‐DT construct along with five biotinylated RNA fragments (F1: 1–1215 nt; F2: 1–590 nt; F3: 1–840 nt; F4: 841–1215 nt; F5: 591–840 nt; F6: full‐length antisense) for pull‐down experiments in breast cancer cell lysates. Deletion mapping analysis revealed that the 591–840 nt region of CD2BP2‐DT is essential for its interaction with YBX1 (Figure 3I). This is consistent with the results expected from the catRAPID website (Figure S3E, Supporting Information). To determine the domain of YBX1 involved in this interaction, we constructed a series of Flag‐tagged truncated YBX1 proteins (Figure 3J). RIP assays with a Flag antibody demonstrated that the P2, P3, and P4 domains specifically interact with CD2BP2‐DT (Figure 3K,L). In addition, the binding of CD2BP2‐DT and YBX1 was simulated by molecular docking, and the specific binding sites were analyzed (Figure 3M).

LncRNAs are known to specifically bind to RBPs, and influence their functions.^[^ ^29^ ^]^ To investigate whether CD2BP2‐DT regulates the expression of YBX1, we assessed its impact on YBX1 levels. The results indicated that the expression of YBX1 remained unchanged following either knockdown or overexpression of CD2BP2‐DT (Figure S3F, Supporting Information). Moreover, we constructed two siRNAs for YBX1 knockdown (si‐YBX1#1 and si‐YBX1#2) and an overexpression plasmid. The efficacy of these constructs was verified by qRT‐PCR and Western blotting experiments (Figure 3N; Figure S3G, Supporting Information). We selected the si‐YBX1 (si‐YBX1#1) exhibiting the most effective knockdown for further experiments. When YBX1 was overexpressed in breast cancer cells with stable CD2BP2‐DT knockdown, the proliferation inhibition caused by CD2BP2‐DT knockdown was partially reversed (Figure 3O,P; Figure S3H,I, Supporting Information). Conversely, YBX1 knockdown suppressed the proliferation‐promoting effect of CD2BP2‐DT overexpression (Figure S3J,K, Supporting Information). These findings indicate that the promotion of breast cancer cell proliferation by CD2BP2‐DT is dependent on YBX1.

### 2.4 CD2BP2‐DT Facilitates the Interaction between YBX1 and CDK1 mRNA

**Figure 4:**
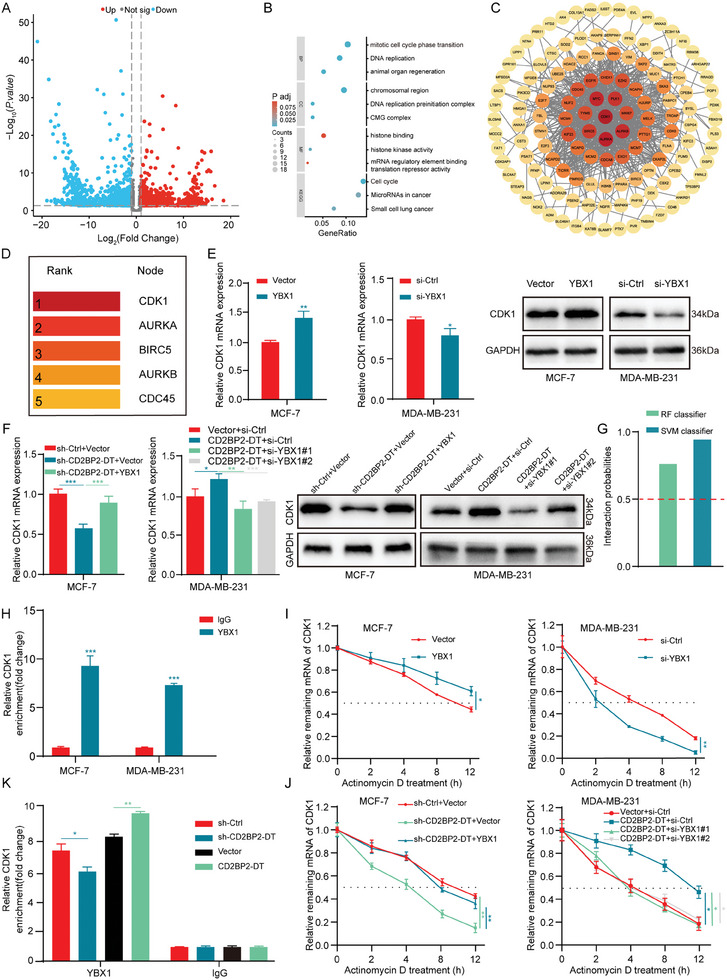
CD2BP2‐DT facilitates the interaction between YBX1 and CDK1 mRNA. A) Volcano plot of the DEGs after CD2BP2‐DT knockdown in breast cancer cells. B) GO and KEGG enrichment analyses were conducted to investigate the functions and pathways associated with potential target genes of CD2BP2‐DT. C) PPI networks of target genes were constructed using Cytoscape software. D) Hub genes were identified using the CytoHubba plugin in Cytoscape software. E) Western blotting and qRT‐PCR analysis demonstrating the expression of CDK1 in breast cancer cells transfected with YBX1 overexpression or knockdown constructs (n = 3). F) qRT‐PCR and Western blotting were used to analyze the effects of YBX1 knockdown or overexpression on CD2BP2‐DT‐mediated CDK1 expression levels (n = 3). G) Prediction of YBX1 binding to CDK1 mRNA using the RPISeq database. H) RIP assay for detection of YBX1 enrichment for CDK1 mRNA in breast cancer cells (n = 3). I) The remaining CDK1 mRNA was detected using qRT‐PCR in breast cancer cells with YBX1 overexpression or knockdown following ActD treatment (n = 3). J) The ActD assay was used to analyze the effects of YBX1 knockdown or overexpression on CD2BP2‐DT‐mediated stability of CDK1 mRNA (n = 3). K) RIP assay using YBX1 antibody in breast cancer cells with CD2BP2‐DT overexpression or knockdown. Enrichment of CDK1 mRNA was analyzed by qRT‐PCR (n = 3). Data in (E), (H), and (K) were calculated by unpaired Student's *t*‐test. Data in (F) were analyzed using one‐way ANOVA test. Data in (I) and (J) were calculated by two‐way ANOVA test. Results are presented as mean ± SD. Significance levels are indicated as *p < 0.05, **p < 0.01, and ***p < 0.001.

In the cytoplasm, YBX1 tends to promote tumor progression by stabilizing mRNA.^[^ ^30^, ^31^, ^32^ ^]^ To elucidate the biological pathways through which the CD2BP2‐DT/YBX1 axis drives breast cancer proliferation and identify its downstream targets, we conducted RNA sequencing after CD2BP2‐DT knockdown, which revealed 4236 DEGs (|log FC| > 0.585, *p* < 0.05) (**Figure** **4**A; Figure S4A, Supporting Information). We further analyzed genes co‐expressed with YBX1 using data from the TCGA database. Additionally, we utilized Cross‐Linking and Immunoprecipitation High‐Throughput Sequencing data from The Encyclopedia of RNA Interactomes database to identify RNAs bound by YBX1. As shown in the Venn diagram, 188 genes were identified as potential targets of the CD2BP2‐DT/YBX1 axis (Figure S4B, Supporting Information). Kyoto Encyclopedia of Genes and Genomes enrichment analysis revealed that the most significantly enriched pathways were primarily involved in the cell cycle. Gene Ontology Biological Process enrichment analysis also indicated a significant association with mitotic cell cycle phase transitions (Figure 4B). Furthermore, we performed a protein‐protein interaction (PPI) network analysis of the 188 potential target genes using the STRING database and visualized the results with Cytoscape software. This analysis identified CDK1 as the most crucial node protein in the interaction network (Figure 4C,D).

CDK1 is overexpressed in breast cancer and functions as a key regulatory factor during the G2/M phase of the cell cycle, thereby promoting tumorigenesis and cancer progression.^[^ ^33^, ^34^ ^]^ Bioinformatics analysis showed that CDK1 is significantly overexpressed in breast cancer (Figure S4C, Supporting Information) and correlates with poor survival prognosis in breast cancer patients (Figure S4D, Supporting Information). We subsequently investigated the regulatory roles of CD2BP2‐DT and YBX1 in CDK1 expression. The results demonstrated that both mRNA and protein levels of CDK1 were altered following knockdown or overexpression of CD2BP2‐DT or YBX1 (Figure 4E; Figure S4E–G, Supporting Information). Rescue experiments demonstrated that the inhibitory effect of stable CD2BP2‐DT knockdown on CDK1 expression was reversed by YBX1 overexpression in breast cancer, while the promoting effect of CD2BP2‐DT overexpression on CDK1 was inhibited by YBX1 knockdown (Figure 4F; Figure S4H, Supporting Information). These findings suggest that CD2BP2‐DT regulates CDK1 expression through a YBX1‐dependent pathway. Research indicates that YBX1 can promote the stability of multiple oncogenic transcripts in breast cancer cells by binding to the 3′UTR region of mRNA.^[^ ^35^ ^]^ Analysis using the Mapping Binding Sites of RNA Binding Proteins database identified several potential binding sites for YBX1 in the 3′UTR region of CDK1 (Figure S4I, Supporting Information). Consistently, analysis using the RNA‐Protein Interaction Prediction database confirmed this binding with high confidence (Figure 4G). Therefore, we speculated that YBX1 enhances mRNA stability by directly binding to CDK1. In the RIP assay, anti‐YBX1 significantly enriched CDK1 mRNA compared to IgG (Figure 4H; Figure S4J, Supporting Information). Actinomycin D (ActD) experiments demonstrated that YBX1 knockdown significantly reduced the stability of CDK1 mRNA, while YBX1 overexpression enhanced CDK1 mRNA stability (Figure 4I; Figure S4K, Supporting Information). Subsequently, we investigated the role of CD2BP2‐DT in the regulation of CDK1 mRNA stability by YBX1. The results of the ActD rescue experiment showed that CDK1 mRNA stability was reduced in breast cancer cells following CD2BP2‐DT knockdown, and this reduction was partially reversed by YBX1 overexpression in the context of CD2BP2‐DT knockdown. Conversely, CDK1 mRNA stability was increased in breast cancer cells after CD2BP2‐DT overexpression, but this increase was inhibited when YBX1 was knocked down during CD2BP2‐DT overexpression (Figure 4J; Figure S4L, Supporting Information). Furthermore, CD2BP2‐DT knockdown decreased the binding of YBX1 to CDK1 mRNA, whereas CD2BP2‐DT overexpression increased their binding, as verified by RIP assays (Figure 4K). In summary, CD2BP2‐DT facilitates the interaction between YBX1 and CDK1 mRNA, thereby enhancing the stability of CDK1 transcripts.

### 2.5 YBX1 Undergoes LLPS in Breast Cancer

**Figure 5:**
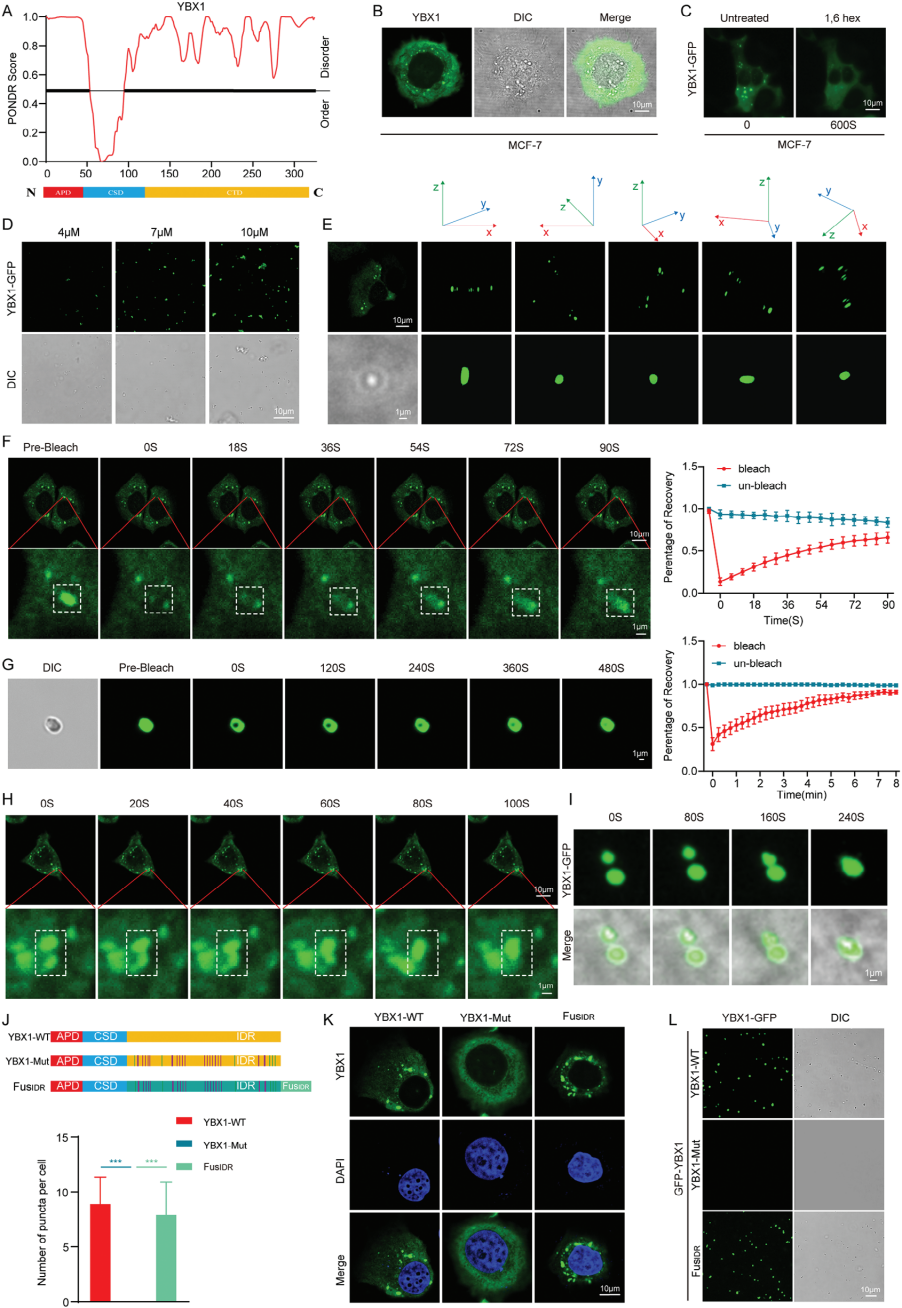
YBX1 undergoes LLPS in breast cancer. A) The IDRs of YBX1 were predicted using the PONDR database. The bottom panel shows a schematic diagram of the YBX1 protein domains. B) Representative images of breast cancer cells transfected with YBX1‐GFP (scale bars, 10 µm). C) Representative images of breast cancer cells transfected with YBX1‐GFP and treated with 1.5% 1,6‐hexanediol (scale bars, 10 µm). D) YBX1‐GFP formed liquid droplets in vitro in a concentration‐dependent manner (scale bars, 10 µm). E) 3D images of YBX1 forming droplet condensates in vivo and in vitro (scale bars, 10 µm, 1 µm). F) FRAP experiments were conducted to assess the recovery of fluorescence in YBX1‐GFP droplets following photobleaching in breast cancer cells (n = 3; scale bars, 10 µm, 1 µm). G) Fluorescence intensity of YBX1‐GFP droplets recovered after bleaching in FRAP assay (n = 3; scale bars, 1 µm). H) Live‐cell imaging of YBX1‐GFP droplet fusion in breast cancer cells (scale bars, 10 µm, 1 µm). I) In vitro phase separation assays demonstrate the fusion of YBX1‐GFP droplets (scale bars, 1 µm). J) Schematic illustration of YBX1 protein and its mutants (WT: Wild Type, Mut: Mutant). K) Representative images of cells transfected with different YBX1 constructs were taken by laser confocal microscopy and statistically analyzed (n = 3; scale bars, 10 µm). L) Representative images of purified YBX1‐WT, YBX1‐Mut, and Fus_IDR_ proteins forming droplets in vitro (scale bars, 10 µm). Data in (J) were analyzed using one‐way ANOVA test. Results are presented as mean ± SD. Significance levels are indicated as ***p < 0.001.

Recent studies have demonstrated that RBPs containing IDRs tend to undergo phase separation, a process that contributes to the stabilization of target gene mRNAs.^[^ ^15^, ^17^, ^20^, ^21^ ^]^ Furthermore, lncRNAs can drive tumorigenesis by promoting the phase separation of RBPs.^[^ ^18^, ^19^ ^]^ As illustrated in Figure 3G, CD2BP2‐DT and YBX1 display a punctate distribution within the cytoplasm. Therefore, we hypothesized that CD2BP2‐DT may regulate CDK1 by enhancing the LLPS of YBX1. Bioinformatics analysis revealed that the C‐terminal Domain (CTD) of YBX1 contains a significant number of IDRs (**Figure** **5**A). Notably, in osteosarcoma cells, the IDRs in the CTD of YBX1 facilitate the occurrence of LLPS.^[^ ^36^ ^]^ Therefore, we investigated whether YBX1 undergoes LLPS in breast cancer cells. We observed the formation of droplet condensates in the cytoplasm of breast cancer cells following transfection with the YBX1‐GFP plasmid (Figure 5B). Notably, treatment with 1,6‐hexanediol resulted in a substantial reduction in the YBX1 condensates (Figure 5C). To further examine YBX1 phase separation, we purified recombinant YBX1‐GFP, which formed droplets in vitro. The formation of these YBX1‐GFP droplets was found to be protein concentration‐dependent (Figure 5D). Additionally, 3D imaging using laser confocal microscopy revealed that YBX1 in the cytoplasm of breast cancer cells, as well as the in vitro purified protein, exhibited a droplet‐like distribution pattern (Figure 5E). The dynamics of these droplets were further investigated using Fluorescence recovery after photobleaching (FRAP). In living cells, YBX1 liquid droplets recovered most of their fluorescence signals within ≈90 s after photobleaching (Figure 5F). In vitro, YBX1‐GFP droplets took ≈480 s to recover most of their fluorescence signals during FRAP analysis (Figure 5G). Further observations revealed that YBX1 liquid droplets exhibited fusion behavior in breast cancer cells (Figure 5H). Similarly, YBX1‐GFP droplets also underwent fusion in vitro (Figure 5I). These results demonstrated that YBX1 proteins can undergo LLPS in the cytoplasm of breast cancer cells, with YBX1 condensates displaying a highly dynamic process of droplet formation both in vitro and in vivo.

To further characterize the phase separation of YBX1 and confirm the necessity of IDRs for YBX1 droplet formation, we constructed three YBX1‐expressing constructs: YBX1‐WT, YBX1‐Mut (RK/G), and Fus_IDR_ (Mut + Fus_IDR_) (Figure 5J). As expected, YBX1‐Mut lost its phase separation ability compared to YBX1‐WT, which was largely restored by Fus_IDR_ (Figure 5K,L). These results highlight the critical role of IDR integrity for YBX1 phase separation in breast cancer cells.

### 2.6 CD2BP2‐DT Promotes YBX1 Phase Separation

**Figure 6:**
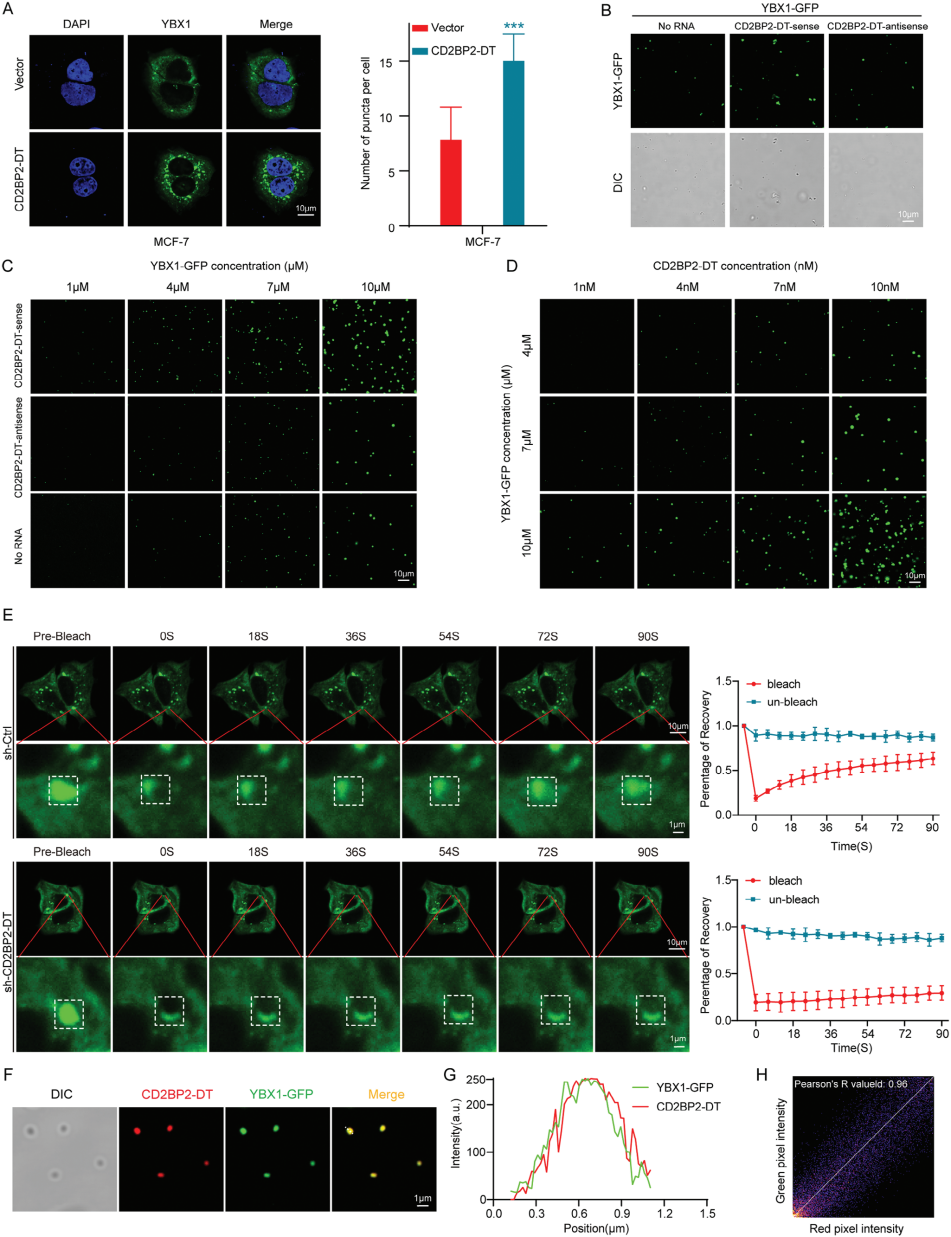
CD2BP2‐DT induces LLPS of YBX1. A) Vectors or CD2BP2‐DT overexpression constructs were transfected into breast cancer cells with YBX1‐GFP overexpression, and the breast cancer cells were subsequently analyzed using confocal microscopy and statistically analyzed (n = 3; scale bars, 10 µm). B) Representative images of YBX1‐GFP in vitro droplet formation with CD2BP2‐DT‐sense, CD2BP2‐DT‐antisense, or No RNA (scale bars, 10 µm). C) Fluorescence microscopy was performed to observe the in vitro droplet formation of different concentrations of YBX1‐GFP in the presence of CD2BP2‐DT‐sense, CD2BP2‐DT‐antisense, or No RNA (scale bars, 10 µm). D) Representative images from in vitro experiments demonstrating the effects of varying amounts of CD2BP2‐DT added to different concentrations of purified YBX1‐GFP protein (scale bars, 10 µm). E) FRAP assay for the analysis of fluorescence recovery following photobleaching of YBX1‐GFP in CD2BP2‐DT knockdown breast cancer cells (n = 3; scale bars, 10 µm, 1 µm). F) Representative images of purified YBX1‐GFP protein co‐localizing with Cy3‐tagged CD2BP2‐DT in droplets (scale bars, 1 µm). G) Fluorescence co‐localization was statistically analyzed using image J software. H) Correlation of green and red fluorescence was further analyzed using ImageJ software. Data in (A) were analyzed using unpaired Student's *t*‐test. Data in (H) were calculated by the Pearson correlation test. Results are presented as mean ± SD. Significance levels are indicated as ***p < 0.001.

We further investigated the role of CD2BP2‐DT in the formation of YBX1 liquid droplet condensates. Fluorescence imaging revealed that overexpression of CD2BP2‐DT significantly enhanced the formation of YBX1 droplets, while knockdown of CD2BP2‐DT markedly inhibited YBX1 droplet formation in breast cancer cells (**Figure** **6**A; Figure S5A, Supporting Information). Additionally, in vitro assays using purified YBX1‐GFP protein and RNA demonstrated that CD2BP2‐DT‐sense promoted the formation of droplets mediated by YBX1‐GFP (Figure 6B). Fluorescence imaging further revealed that CD2BP2‐DT drives YBX1 to form numerous droplets in a dose‐dependent manner (Figure 6C,D). Furthermore, knockdown of CD2BP2‐DT significantly inhibited the fluorescence recovery following photobleaching of YBX1 droplets within cells (Figure 6E). Subsequently, YBX1‐GFP was mixed with Cyanine 3‐labeled CD2BP2‐DT‐sense and analyzed by fluorescence imaging. As anticipated, CD2BP2‐DT completely colocalized with YBX1 within the droplets (Figure 6F). Next, we examined the overlapping regions and observed a strong association between the signal intensities of CD2BP2‐DT and YBX1 (Figure 6G). Further analysis using distinct colocalization algorithms revealed a high correlation between the signals (Pearson's R = 0.96, *p* < 0.05) (Figure 6H). Altogether, these findings indicate that CD2BP2‐DT promotes the formation of YBX1 LLPS.

### 2.7 YBX1 Phase Separation Promotes the Stabilization of CDK1 mRNA

**Figure 7:**
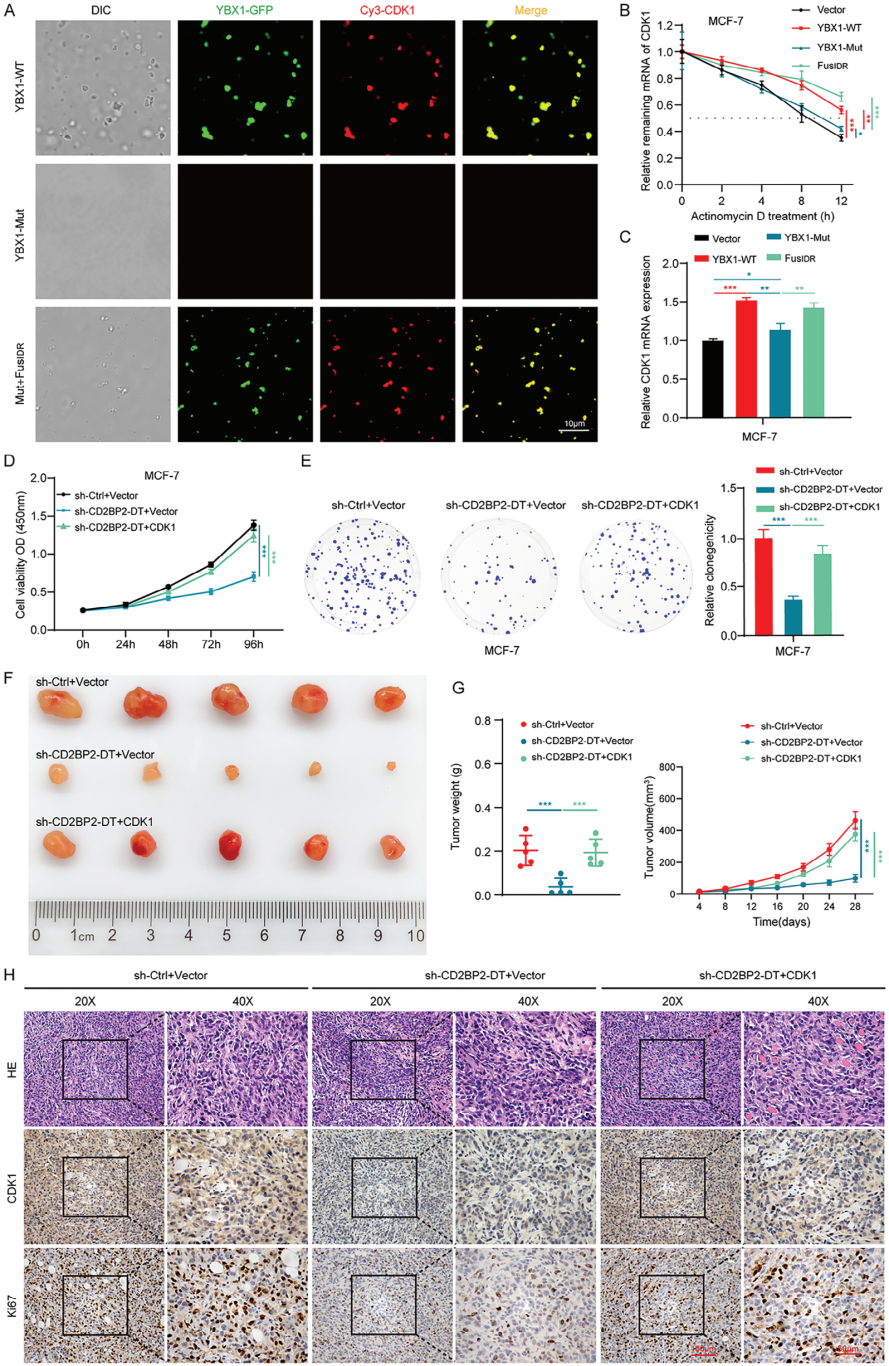
CD2BP2‐DT‐mediated YBX1 phase separation promotes the stability of CDK1 mRNA and CD2BP2‐DT/CDK1 axis promotes proliferation of breast cancer cells. A) Representative images from in vitro phase separation experiments involving purified YBX1‐WT, YBX1‐Mut, and FusIDR proteins complexed with Cy3‐tagged CDK1 mRNA (scale bars, 10 µm). B) ActD assay to detect the degradation rate of CDK1 mRNA in breast cancer cells transfected with YBX1‐WT, YBX1‐Mut, or Fus_IDR_ (n = 3). C) The expression levels of CDK1 in breast cancer cells transfected with YBX1‐WT, YBX1‐Mut, or FusIDR were assessed using qRT‐PCR (n = 3). D, E) CCK‐8 (D) and colony formation (E) assays were used to analyze the effects of CDK1 overexpression on CD2BP2‐DT‐mediated breast cancer cell proliferation (n = 3). F) Image of subcutaneous xenograft tumors in nude mice from various experimental treatment groups. G) Growth volume curve and tumor mass analysis of tumors (n = 5). H) Representative images of HE staining, and IHC staining for CDK1 and Ki67, in xenograft tumor tissues from different experimental groups (n = 5; scale bars, 100 µm, 50 µm). Data in (B), (D), and (G) were calculated by two‐way ANOVA test. Data in (C), (E), and (G) were analyzed using one‐way ANOVA test. Results are presented as mean ± SD. Significance levels are indicated as *p < 0.05, **p < 0.01, and ***p < 0.001.

Recent studies have highlighted the role of RBP phase separation in stabilizing target gene mRNAs.^[^ ^20^, ^21^ ^]^ Given that CD2BP2‐DT binds to YBX1 and promotes its phase separation, we hypothesize that YBX1 enhances the stability of CDK1 mRNA through condensate formation via LLPS. To evaluate this hypothesis, we mixed purified YBX1 proteins (YBX1‐WT, YBX1‐Mut, and Fus_IDR_) with Cy3‐labeled CDK1 mRNA and performed fluorescence imaging. Our results demonstrated that CDK1 mRNA was enriched within YBX1‐WT condensates, whereas YBX1‐Mut failed to recruit CDK1 mRNA (**Figure** **7**A). However, the introduction of Fus_IDR_ largely restored this recruitment capability. In addition, treatment with ActD and qRT‐PCR experiments demonstrated that YBX1‐WT or FusIDR enhanced the stability and expression levels of CDK1 mRNA, whereas no such effect was observed for YBX1‐Mut (Figure 7B,C). Collectively, these findings suggest that CD2BP2‐DT‐mediated phase separation of YBX1 enhances the stability and expression of CDK1 mRNA.

### 2.8 CDK1 is Involved in CD2BP2‐DT Induced Proliferation in Breast Cancer

Next, we investigated the role of the CD2BP2‐DT/CDK1 regulatory axis in promoting breast cancer proliferation. First, we confirmed the knockdown efficiency of CDK1 using qRT‐PCR and Western blotting (Figure S6A, Supporting Information). Cellular experiments revealed that overexpression of CDK1 substantially reversed the proliferation inhibition observed upon CD2BP2‐DT knockdown in breast cancer cells (Figure 7D,E; Figure S6B,C, Supporting Information). Conversely, CDK1 knockdown effectively attenuated the proliferative effect of CD2BP2‐DT overexpression in breast cancer cells (Figure S6D, E, Supporting Information). In addition, tumor xenograft experiments showed that knockdown of CD2BP2‐DT markedly inhibited breast cancer cell proliferation and decreased tumor volume and weight in vivo. This effect was reversed by overexpression of CDK1 (Figure 7F,G). IHC staining revealed that CD2BP2‐DT knockdown decreased CDK1 protein levels and inhibited Ki67 expression. Conversely, CDK1 overexpression restored CDK1 levels and enhanced Ki67 staining (Figure 7H; Figure S6F, Supporting Information). These findings indicate that the promotion of breast cancer cell proliferation by CD2BP2‐DT is dependent on CDK1.

CDK1 is a member of the cell cycle‐dependent kinase family, and its function is largely dependent on kinase activity.^[^ ^37^ ^]^ We further investigated the alterations in CDK1 phosphorylation at specific sites (Thr14, Tyr15, Thr161) following the knockdown or overexpression of CD2BP2‐DT. The results indicated that CD2BP2‐DT significantly influences the expression level of CDK1^Thr161^, primarily by regulating the total CDK1 protein levels (Figure S7A, Supporting Information). Additionally, we examined the impact of CD2BP2‐DT on the cell cycle. Knockdown of CD2BP2‐DT led to G2/M phase arrest, while overexpression of CD2BP2‐DT facilitated the transition through G2/M phase (Figure S7B, Supporting Information). In conclusion, CD2BP2‐DT promotes the expression of total CDK1 protein and markedly upregulates CDK1^Thr161^ levels, thereby facilitating the G2/M phase transition in breast cancer cells.

### 2.9 N4‐Acetylcytidine Modification is Involved in the Upregulation of CD2BP2‐DT in Breast Cancer

**Figure 8:**
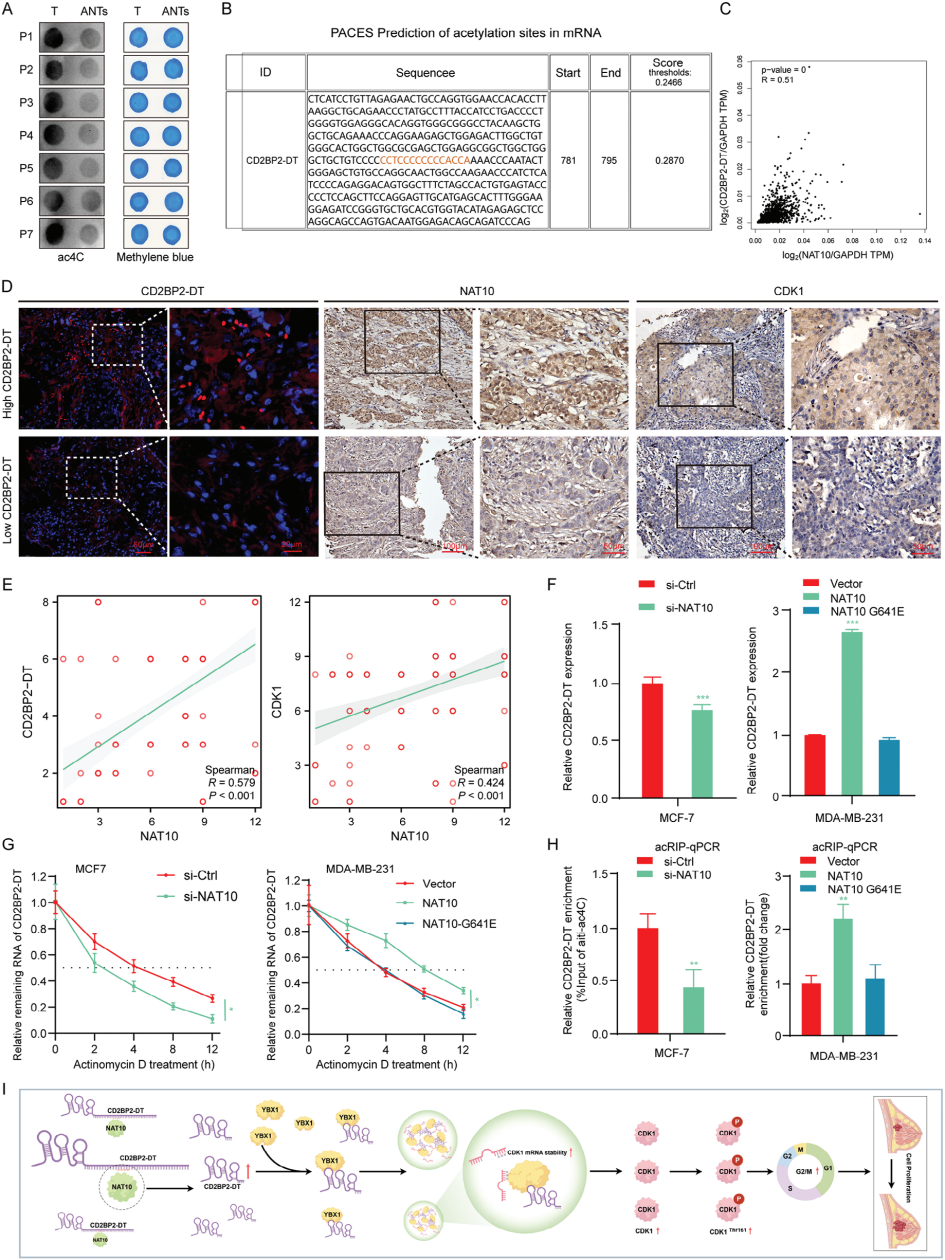
N4‐acetylcytidine modification is involved in the upregulation of CD2BP2‐DT in breast cancer. A) Dot blot assays are employed to assess acetylation levels in breast cancer. B) An online database was employed to predict potential ac4C modification sites within the lncRNA CD2BP2‐DT. C) The TCGA database was utilized to analyze the correlation between NAT10 expression and the lncRNA CD2BP2‐DT in breast cancer. D) Representative images of FISH and IHC staining for TMA (scale bars, 100 µm, 50 µm, 20 µm). E) Correlation analysis of NAT10 with CD2BP2‐DT and CDK1 in TMA (n = 114). F) The expression of CD2BP2‐DT was assessed using qRT‐PCR following the knockdown or overexpression of NAT10 (n = 3). G) The degradation rate of CD2BP2‐DT was measured in breast cancer cells transfected with si‐NAT10 or a NAT10 overexpression plasmid using the ActD method (n = 3). H) The ac4C‐RIP assay was conducted to evaluate the level of ac4C modification on CD2BP2‐DT in breast cancer cells transfected with either si‐NAT10, NAT10 G641E mutant, or a NAT10 overexpression plasmid (n = 3). I) Proposed model for the CD2BP2‐DT‐mediated proliferation of breast cancer and the image is drawn by Figdraw. Data in (C) and (E) were calculated by the Spearman correlation test. Data in (F) and (H) were calculated by unpaired Student's *t*‐test and one‐way ANOVA test. Data in (G) were calculated by two‐way ANOVA test. Results are presented as mean ± SD. Significance levels are indicated as *p < 0.05, **p < 0.01, and ***p < 0.001.

However, the mechanisms driving the upregulation of the lncRNA CD2BP2‐DT remain unclear. Ac4C, a type of RNA epigenetic modification, has been reported to play a critical role in the regulation of mRNA stability.^[^ ^38^, ^39^ ^]^ Emerging evidence suggests that the ac4C modification also influences the stability and expression of lncRNAs in cancer.^[^ ^40^, ^41^ ^]^ To explore the expression and functional significance of ac4C modifications in breast cancer, RNA ac4C levels were assessed via dot blot analysis in seven pairs of breast cancer and ANTs. The results revealed a notable enrichment of ac4C modifications in breast cancer tissues compared to their adjacent counterparts (**Figure** **8**A). Bioinformatics analyses indicated that CD2BP2‐DT contains potential ac4C modification sites (Figure 8B). NAT10 is the sole enzyme known to catalyze RNA acetylation. Correlation analysis revealed a positive association between NAT10 expression and CD2BP2‐DT across various solid tumors (Figure S8A, Supporting Information), suggesting that NAT10‐mediated ac4C modification plays a widespread role in regulating CD2BP2‐DT. In the present study, we further analyzed the correlation between NAT10, CD2BP2‐DT, and CDK1 using bioinformatic databases and IHC on TMA in breast cancer. The results consistently demonstrated that NAT10 expression in breast cancer tissues positively correlated with CD2BP2‐DT and CDK1 levels, suggesting that NAT10 acts as an upstream regulator of the CD2BP2‐DT/CDK1 signaling axis (Figure 8C–E; Figure S8B, Supporting Information). Subsequently, we synthesized siRNA for NAT10 knockdown, a plasmid for NAT10 overexpression, and a plasmid containing a mutation in the active site of acetyltransferase (G641E). The efficiency of both knockdown and overexpression was verified using qRT‐PCR and Western blotting (Figure S8C,D, Supporting Information). Knockdown of NAT10 results in a reduction of CD2BP2‐DT RNA levels, whereas overexpression of NAT10 leads to increased CD2BP2‐DT expression. However, the NAT10 G641E mutant failed to regulate CD2BP2‐DT expression (Figure 8F; Figure S8E, Supporting Information). These findings suggest that NAT10‐mediated RNA acetylation is critical for modulating CD2BP2‐DT expression. Given the importance of RNA epigenetic modifications in stabilizing transcripts, we performed ActD assays to assess the impact of NAT10‐mediated ac4C modification on CD2BP2‐DT stability. The results revealed that knockdown of NAT10 significantly reduced the stability of CD2BP2‐DT, whereas overexpression of NAT10 enhanced its stability. In contrast, the NAT10 G641E mutant had no effect on CD2BP2‐DT stability (Figure 8G; Figure S8F, Supporting Information). Furthermore, the ac4C‐RIP analysis demonstrated that CD2BP2‐DT enrichment in anti‐ac4C precipitates was significantly reduced following NAT10 knockdown. Conversely, NAT10 overexpression increased CD2BP2‐DT enrichment (Figure 8H; Figure S8G, Supporting Information). These findings underscore the role of NAT10‐mediated ac4C modification in stabilizing CD2BP2‐DT, further highlighting its regulatory function in breast cancer pathogenesis.

## 3 Discussion

More than 68% of the genes in the human genome are classified as lncRNAs, which are increasingly recognized as key regulators of tissue physiology and disease processes.^[^ ^42^ ^]^ Therefore, targeting endogenous non‐coding RNAs (ncRNAs) offers significant potential for the treatment of a number of diseases. Currently, several RNA‐based therapies are in phase II or III of clinical development, indicating the imminent clinical application of lncRNA‐based treatments.^[^ ^43^ ^]^ The identification of novel lncRNAs, elucidation of their functions, and exploration of their associations with various cancer subtypes have ushered in a transformative paradigm in cancer research, positioning these approaches as essential strategies in cancer therapy.^[^ ^44^ ^]^ In this study, we identified a novel lncRNA, CD2BP2‐DT, through analysis of publicly available datasets, which revealed its elevated expression in multiple cancers, including breast cancer. This finding was further validated in breast cancer tissues and cell lines. Additionally, our analysis demonstrated a positive correlation between increased CD2BP2‐DT expression and larger tumor size in breast cancer patients, suggesting a significant role for CD2BP2‐DT in cancer progression. Both in vivo and in vitro experiments confirmed that CD2BP2‐DT significantly boosts breast cancer cell proliferation, highlighting its crucial role in this process.

RBPs play a crucial role in the pathogenesis of breast cancer by influencing tumor cell proliferation, survival, and response to therapy through interactions with mRNAs, proteins, DNA, ncRNAs, and various signaling pathways.^[^ ^45^ ^]^ Targeting these interactions presents a promising therapeutic approach for cancer treatment. With advances in proteomics and epigenetics, an increasing number of RBPs have been identified, and the interaction between lncRNAs and RBPs has garnered significant attention.^[^ ^23^ ^]^ Previous studies have primarily focused on the function of lncRNAs as decoys, scaffolds, or guides for RBPs. These interactions significantly influence the modification, stability, localization, and activity of their binding partners.^[^ ^23^ ^]^ More recently, studies have highlighted an emerging role for lncRNAs as molecular scaffolds actively involved in regulating phase separation.^[^ ^18^ ^]^ This expanding network of lncRNA‐RBP interactions provides a novel framework for understanding the mechanisms underlying cancer initiation and progression. For example, Gao et al. identified the lncRNA MTAR1, which promotes cancer progression by enhancing the post‐transcriptional regulation of c‐MYC via the mediation of PABP1/IGF2BPs LLPS.^[^ ^46^ ^]^ However, the mechanism underlying lncRNA‐mediated phase separation of RBPs in tumorigenesis is still not fully understood. Our investigation reveals that YBX1 undergoes LLPS in the cytoplasm of breast cancer cells. Furthermore, overexpression of CD2BP2‐DT significantly enhances the formation of YBX1 droplets both in vivo and in vitro. These findings elucidate the mechanism by which CD2BP2‐DT interacts with YBX1 and facilitates its phase separation in breast cancer. Nonetheless, lncRNAs may also function as molecular sponges by binding to microRNAs.^[^ ^47^ ^]^ Whether CD2BP2‐DT possesses additional regulatory functions remains to be investigated.

In 2009, Hyman's team elucidated the phenomenon of phase separation by demonstrating that P granules exhibit liquid‐like properties.^[^ ^48^ ^]^ Membraneless organelles formed through LLPS are essential to a variety of biological processes, including chromatin organization, genome stability, DNA damage response and repair, transcription, and signal transduction. Dysregulation of these processes is a critical factor in the initiation and progression of cancer.^[^ ^49^ ^]^ Liu et al. demonstrated that the LLPS of nuclear YBX1, enhanced by circASH2, accelerates the decay of TPM4 transcripts.^[^ ^50^ ^]^ Additionally, MELTF‐AS1 can drive the phase separation of YBX1, thereby activating ANXA8 transcription and promoting tumorigenesis in non‐small cell lung cancer.^[^ ^51^ ^]^ These studies suggest that the LLPS of YBX1 within the nucleus is essential for transcriptional regulation. However, the function of phase separation of YBX1 in the cytoplasm remains unclear. In our study, we demonstrate that CD2BP2‐DT enhances the stability of CDK1 mRNA via YBX1, which is achieved by the condensation of mRNA mediated by YBX1 phase separation. Our findings uncover a novel mechanism that diverges from the well‐established role of YBX1 in transcriptional regulation via nuclear LLPS, which may be related to tumor heterogeneity. However, further investigation is warranted to elucidate the role of the phase‐separated environment created by YBX1 in regulating translation, post‐translational modifications, and other aspects within breast cancer cells.

Maintaining proliferation signals, evading growth inhibitors, resisting cell death, and achieving replicative immortality are key hallmarks of cancer.^[^ ^52^ ^]^ The cell cycle, a series of tightly regulated events, drives cellular growth and proliferation.^[^ ^53^ ^]^ Studies have demonstrated that CDK1 is a crucial regulator of the cell cycle in most mammalian cells.^[^ ^54^ ^]^ Dysregulation of CDK1 can lead to abnormal tumor proliferation and chromosomal instability.^[^ ^33^ ^]^ Recently, Menon et al. reported that overexpression of CDK1 in melanoma cells promotes tumorigenesis in human melanoma.^[^ ^55^ ^]^ Additionally, Ren et al. found that high CDK1 expression drives the progression of adrenocortical carcinoma by regulating the G2/M phase transition.^[^ ^56^ ^]^ Over the past few decades, a variety of selective inhibitors and pan‐inhibitors targeting CDK1 have been developed. Notably, several small molecule inhibitors aimed at CDK1 have progressed to clinical trials, highlighting the potential of CDK1 as a viable target for cancer treatment.^[^ ^57^ ^]^ In this study, we conducted a comprehensive evaluation of CDK1 expression in breast cancer and its association with patient survival prognosis. Our findings indicate that elevated levels of CDK1 expression are correlated with poor prognostic outcomes in patients diagnosed with breast cancer. Further experiments demonstrated that CD2BP2‐DT enhances breast cancer cell proliferation by modulating the expression of CDK1. These findings indicate that targeting CDK1 via the CD2BP2‐DT/YBX1 axis may represent a promising anticancer strategy for breast cancer and warrants further investigation.

Epigenetic modifications of RNA, including N⁶‐Methyladenosine (m6A), 5‐Methylcytosine (m5C), N¹‐Methyladenosine (m1A), ac4C, and others, are prevalent across eukaryotic systems and exert critical influences on RNA structure and function.^[^ ^58^ ^]^ Among these, ac4C has recently emerged as a key regulator of RNA stability and translation.^[^ ^59^ ^]^ NAT10, the only known RNA acetyltransferase that catalyzes ac4C modification, has been implicated in tumorigenesis by increasing ac4C levels, highlighting its crucial role in cancer progression. Li et al. demonstrated that NAT10 stabilizes HMGA1 mRNA through acetylation, thereby promoting cell cycle progression and enhancing prostate cancer cell proliferation.^[^ ^38^ ^]^ However, the dysregulation of ac4C‐modified non‐coding RNAs in tumors remains largely unexplored. Our findings indicate elevated levels of ac4C modification in breast cancer tissues. Furthermore, ac4C modification of CD2BP2‐DT enhances its RNA stability and expression. Elucidating the regulatory pathways and molecular drivers responsible for the aberrant expression of CD2BP2‐DT is crucial for understanding its oncogenic role and identifying novel therapeutic targets. However, the presence of additional RNA modifications and regulatory mechanisms influencing the expression of CD2BP2‐DT in breast cancer warrants further investigation.

LLPS, driven by IDRs and RNA‐protein interactions, facilitates the formation of dynamic biomolecular aggregates that are critical for regulating critical biological processes. The LLPS capability of YBX1 enables it to orchestrate the assembly of transcriptional and signaling complexes within these aggregates, positioning it as a promising therapeutic target. However, the extensive IDR characteristics of YBX1 present considerable challenges for conventional structure‐based drug discovery and design. Recent studies have shown that droplets formed through phase separation can dilute the effective concentration of anti‐cancer drugs, such as tamoxifen, contributing to drug resistance in breast cancer cells.^[^ ^60^ ^]^ This finding underscores the potential of small molecules to disrupt phase‐separated complexes as a promising therapeutic strategy. Emerging evidence highlights the considerable promise of phase separation‐based approaches in targeting non‐structured proteins. With rapid advancements in bioinformatics technologies, the integration of machine learning and pharmacogenomics has emerged as an innovative method for identifying drugs and biomarkers for breast cancer.^[^ ^61^, ^62^, ^63^, ^64^ ^]^ Through this technological convergence, research centered on LLPS is anticipated to substantially enhance the development of anti‐cancer drugs. By focusing on key molecules such as CD2BP2‐DT and YBX1, we broaden the horizons of cancer treatment while highlighting the groundbreaking application of LLPS technology in addressing previously undruggable proteins. In addition, small molecule inhibitors targeting NAT10 have been developed. These inhibitors significantly reduce RNA acetylation levels in cells, positioning NAT10 as a promising candidate for cancer therapy. Targeting NAT10 activity is a promising approach to inhibit the CD2BP2‐DT/YBX1 pathway. Combining NAT10 inhibition with direct targeting of the CD2BP2‐DT/YBX1 signaling pathway provides a multifaceted strategy to suppress tumor cell proliferation and survival, thereby potentially enhancing therapeutic efficacy.

In conclusion, our study demonstrates that NAT10‐mediated ac4C modification enhances the stability of the lncRNA CD2BP2‐DT. CD2BP2‐DT mediates the phase separation of YBX1, thereby stabilizing CDK1 mRNA and promoting the proliferation of breast cancer cells (Figure 8I). These findings indicate that CD2BP2‐DT represents a promising therapeutic target for breast cancer treatment.

## 4 Experimental Section

### Clinical Specimens

A total of 114 pairs of paraffin‐embedded breast cancer tissues and corresponding ANTs were collected for TMA analysis. In addition, 7 pairs of frozen tissues were collected for ac4C dot blot. The study protocol received approval from the Medical Ethics Committee of the Second Affiliated Hospital of Harbin Medical University (YJSKY2023‐170), and all participants gave their informed consent to be included in the study.

### Cell Lines and Culture

The normal human mammary epithelial cell line (MCF‐10A) and the human breast cancer cell lines were obtained from the National Collection of Authenticated Cell Cultures (Shanghai, China). T‐47D cells were cultured in RPMI 1640 medium with 10% fetal bovine serum (Gibco, MA, USA), while other breast cancer cell lines were grown in DMEM. MCF‐10A cells were cultured in a specialized epithelial culture medium (Procell, Wuhan, China). All cell lines were tested for mycoplasma contamination before use to ensure they were free of infection.

### Cell Transfection

siRNAs targeting CD2BP2‐DT (si‐CD2BP2‐DT#1, si‐CD2BP2‐DT#2), YBX1 (si‐YBX1#1, si‐YBX1#2), CDK1 (si‐CDK1), NAT10 (si‐NAT10) and a control (si‐Ctrl) were obtained from Gene Pharma Technology (Shanghai, China). The overexpression plasmid was constructed using the pcDNA3.1 vector (Sangon Biotech, Shanghai, China). The siRNAs and plasmids were transfected into breast cancer cells using jetPRIME Polyplus Transfection reagent (Polyplus‐Transfection, Illkirch, France) according to the manufacturer's instructions. Lentiviruses were obtained from Gene Pharma Technology (Shanghai, China) and transfected into breast cancer cells following the manufacturer's instructions, with puromycin used for cell selection. The sequences of the oligonucleotides are provided in Table S2, Supporting Information.

### Cell Counting Kit‐8 Assay

The treated cells were seeded in a 96‐well plate. At the specific time points, the complete medium was discarded, and 10 µL of CCK‐8 solution (APExBIO, Houston, USA) along with 100 µL of serum‐free medium were added to each well. The cells were then incubated for an additional 2 h, and absorbance was measured at 450 nm in the dark.

### Colony Formation Assay

Transfected breast cancer cells were seeded in 6‐well plates and cultured for a specified period. The cells were then fixed with 4% paraformaldehyde at room temperature for 20 min and stained with 0.3% crystal violet for 15 min. After washing, the colonies were counted and analyzed.

### 5‐Ethynyl‐2′‐Deoxyuridine Incorporation Assay

Transfected breast cancer cells were seeded into 96‐well plates. After 12 h, EdU labeling and Apollo staining were performed using the Cell‐Light EdU Apollo In Vitro Kit (RIBOBIO, Guangzhou, China). Fluorescence detection was then carried out using an inverted fluorescence microscope (Olympus, Tokyo, Japan).

### Quantitative Real‐Time PCR

Total RNA was isolated from breast cancer cells using the RNA Isolater Total RNA Extraction Reagent (Vazyme, Nanjing, China). RNA from the nuclear and cytoplasmic fractions of breast cancer cell lines was isolated using the PARIS Kit (Life Technologies, Austin, Texas, USA). The RNA was reverse transcribed into cDNA using the HiScript II Q RT SuperMix (Vazyme, Nanjing, China). Amplification was carried out using the ChamQ SYBR qPCR Master Mix (Vazyme, Nanjing, China). GAPDH was used as an internal control. The sequences of the primers used are listed in Table S3, Supporting Information.

### Western Blotting

Cell proteins were extracted using a lysis buffer containing RIPA and PMSF, and protein concentration was determined using a BCA Protein Concentration Assay Kit (Beyotime, Shanghai, China). The extracted proteins were electrophoresed on an SDS‐PAGE gel and transferred to a PVDF membrane (Millipore, MA, USA). The membrane was blocked with 5% skim milk and incubated overnight at 4 °C with primary antibodies against YBX1 (1:5000, Proteintech, 20339‐1‐AP), GAPDH (1:50 000, Proteintech 60004‐1‐Ig), CDK1^Thr161^ (1:200, ABclonal, AP0324), CDK1^Tyr15^ (1:200, Santa Cruz sc‐136014), CDK1^Thr14^ (1:1000, ABclonal, AP1465), CDK1 (1:1000, Santa Cruz, sc‐54), NAT10 (1:5000, Proteintech, 13365‐1‐AP) and Flag (1:10 000, Proteintech, 20543‐1‐AP). After incubation with secondary antibodies at room temperature, protein detection was performed using Chemistar High‐sig ECL Western Blot Substrate (Tanon, Shanghai, China).

### RNA Immunoprecipitation‐qRT‐PCR

According to the instructions of the Magna RNA‐Binding Protein Immunoprecipitation Kit (Millipore, MA, USA), the antibody was mixed with protein A/G magnetic beads (MCE, New Jersey, USA) to form antibody‐magnetic bead complexes. These complexes were added to cell lysates and incubated overnight. Afterward, the complexes were treated with proteinase K to release the immunoprecipitated RNA, which was then extracted and analyzed by qRT‐PCR analysis.

### Fluorescence In Situ Hybridization and Immunofluorescence Staining

The lncRNA CD2BP2‐DT probe was designed and synthesized by RIBOBIO (Guangzhou, China). FISH was performed using the Ribo FISH Kit (RIBOBIO, Guangzhou, China). Cells were seeded onto cell slides in 24‐well plates, fixed, and permeabilization before being treated with the CD2BP2‐DT probe. After blocking with Bovine Serum Albumin (Solarbio, Beijing, China), the cells were incubated overnight with the YBX1 antibody (1:200, Proteintech, 20339‐1‐AP). DAPI was used to stain the nucleus. Representative images were captured using a laser confocal microscope (CLSM, Leica STELLARIS 5, Germany).

Additionally, the expression level of CD2BP2‐DT in TMA was evaluated by FISH staining. The total score was calculated by multiplying the staining intensity score by the percentage of positively stained cells and was used to categorize breast cancer samples in the TMA into low and high CD2BP2‐DT expression groups. The scoring criteria were consistent with those used in previous studies.^[^ ^65^ ^]^

### Fluorescence Recovery After Photobleaching Assay

In vivo and in vitro FRAP experiments were conducted using a confocal laser microscope. A suitable region of interest was selected and photobleached 5–8 times using a 488 nm laser pulse. The bleached area was then imaged to record the recovery.

### Protein Purification

YBX1 protein with a GFP tag was expressed in BL21 (DE3) E. coli cells. Protein expression was induced by adding 1 mM Isopropyl β‐D‐1‐thiogalactopyranoside (IPTG) when the bacterial culture reached the mid‐log phase (OD600 0.5‐0.7). His‐tagged YBX1 protein was purified using a commercial His‐tag Protein Purification Kit (Beyotime, Shanghai, China), according to the manufacturer's protocol. The purified proteins were snap‐frozen and stored at −80 °C for subsequent experiments.

### Actinomycin D

Breast cancer cells were treated with 5 µg mL^−1^ Actinomycin D (MCE, New Jersey, USA). The cells were collected at designated time points, and after RNA extraction, the relative residual amount and stability of CDK1 mRNA were assessed by qRT‐PCR.

### Chromatin Isolation by RNA Purification

The ChIRP probe was obtained from RIBOBIO. The CD2BP2‐DT ChIRP probe was added to the prepared cell lysate and incubated for 4 h. BeyoMag Streptavidin Magnetic Beads (Beyotime, Shanghai, China) were then added according to the reagent instructions and incubated for 2 h. The beads were washed and eluted, and the eluted protein samples were subjected to silver staining after Western blotting. Differential bands were excised and analyzed by MS spectrometry (LC‐Bio Technology, Hangzhou, China).

### RNA ac4C Dot Blot

Freshly extracted RNA was heated to 65 °C for 5 min and then immediately placed on ice. RNA at a concentration of 100 ng µL^−1^ was spotted onto a nitrocellulose membrane (Pall Corporation, Washington, USA), crosslinked by UV for 1 h, and blocked with 5% skim milk. The membrane was incubated overnight at 4 °C with the primary antibody (1:500, Abcam, ab252215), followed by a 1‐h incubation at room temperature with the secondary antibody. After washing, the membrane was developed and stained.

### In Vitro Transcription Assays and Biotinylated RNA Pull‐Down

DNA containing the T7 promoter was obtained by PCR amplification, followed by agarose gel electrophoresis and gel recovery using the TIANgel Purification Kit (TIANGEN BIOTECH, Beijing, China) to obtain purified DNA. The purified DNA was incubated with T7 RNA polymerase (Thermo Fisher Scientific, MA, USA), RNase inhibitor, and a biotin RNA labeling mixture or Cy3‐UTP in a PCR instrument at 37 °C for 2 h to obtain in vitro transcribed biotin or Cy3‐labeled RNA. The RNA was then purified using the RNAclean Kit (TIANGEN BIOTECH, Beijing, China) to obtain pure RNA.

The biotin‐labeled RNA probe was added to the prepared cell lysate for incubation, followed by the addition of BeyoMag Streptavidin Magnetic Beads (Beyotime, Shanghai, China) according to the reagent instructions, and incubated for 1 h. The magnetic beads were washed and eluted, and the eluted protein samples were subjected to Western blotting.

### Droplet Assay

In vitro phase separation assays were performed in a physiological LLPS buffer (15 mM NaCl, 20 mM Tris‐HCl, pH 7.5, 5 mM KH_2_PO_4_, 1.5 mM MgCl_2_, 130 mM KCl, and 1 mg mL^−1^ BSA) with PEG8000. For droplet analysis, droplets were added to a confocal dish with a chamber, and imaging analysis was performed using a laser confocal microscope. For co‐phase separation assay, in vitro transcribed lncRNA CD2BP2‐DT or CDK1 mRNA was gently mixed with purified YBX1 protein and incubated for 5 min, followed by imaging analysis using a laser confocal microscope.

### Bioinformatics Analysis

Bioinformatics analyses were performed using publicly available datasets, including tumor cell line expression data from the CCLE database. The coding potential of CD2BP2‐DT was evaluated using CPAT and CPC2, while its subcellular localization was predicted using lncATLAS. The interaction between CD2BP2‐DT and YBX1 was predicted by catRAPID, and the IDRs of YBX1 were analyzed using the PONDR database. Acetylation modification sites on CD2BP2‐DT were predicted using the PACES database, and the binding potential of YBX1 to CDK1 was assessed using RBPmap and RPISeq.

### Rapid‐Amplification of cDNA Ends

5′and 3′RACE analyses were performed according to the instructions of the HiScript‐TS 5′/3′ RACE Kit (Vazyme, Nanjing, China). Specific primers for lncRNA CD2BP2‐DT were designed and synthesized for nested PCR reactions. The PCR products were purified for Sanger sequencing (Sangon Biotech, Shanghai, China).

### RNA Transcriptome Sequencing

Total RNA was extracted from control and CD2BP2‐DT knockdown cells using RNA Isolater Total RNA Extraction Reagent. Sequencing was performed by Sangon Biotech (Shanghai, China).

### Protein‐Protein Interaction Network Construction

The PPI network was constructed using Cytoscape software and the STRING database. Gene interaction data were visualized, with nodes representing proteins and edges indicating their interactions. Node colors reflected degree values and interaction strength, with darker colors indicating higher connectivity and importance. The Maximum Clique Centrality (MCC) algorithm in the CytoHubba plugin was used to identify hub genes. Node color intensity correlates with centrality; a darker red hue indicates higher centrality scores and significance.

### Xenograft Mouse Model

Female BALB/c nude mice, aged 4 to 6 weeks, were obtained from Beijing Vital River Laboratory Animal Technology Co., Ltd. (Beijing, China). Breast cancer cells were injected subcutaneously into the axilla of the mice to establish a xenograft tumor model. Tumor size was measured periodically, and tumor volume was calculated using the formula: V = length × width^2^ × 0.5. At the end of the experiment, subcutaneous tumors were excised for Hematoxylin‐eosin staining (HE) and IHC staining. Ethical approval for all animal experiments was granted by the Medical Ethics Committee of the Second Affiliated Hospital of Harbin Medical University (YJSDW2023‐078).

### Hematoxylin‐Eosin Staining and Immunohistochemistry

HE staining was performed using the hematoxylin‐eosin staining solution. IHC was conducted following the manufacturer's instructions for the streptavidin‐peroxidase (SP) Kit (Zhongshan Biotech, Beijing, China). Antibodies used for IHC included CDK1 (1:300, Santa Cruz, sc‐54), CD133 (1:700, Cell Signaling Technology, 86781S), CD44 (1:300, Cell Signaling Technology, 37259S), SOX2 (1:100, Proteintech, 11064‐1‐AP), NAT10 (1:1000, Proteintech, 13365‐1‐AP), and Ki67 (1:5000, Proteintech, 27309‐1‐AP). Images of HE and IHC staining were captured using an Olympus microscope (Tokyo, Japan). The staining scores were assessed according to criteria established in previous studies.^[^ ^65^ ^]^ The IHC analyses were conducted by two experienced pathologists who were blinded to the target gene status. They independently assessed both the staining intensity and the percentage of positive cells for scoring purposes.

### Statistical Analysis

Data are expressed as the mean ± standard deviation (SD). Statistical significance was defined as *p* < 0.05. Differences between groups were analyzed using unpaired Student's *t*‐test or analysis of variance (ANOVA). The sample size (n) for each statistical analysis is provided in the figure legends. Overall survival was analyzed using the log‐rank test and Kaplan‐Meier method. Correlations were evaluated using Pearson's or Spearman's correlation coefficient. The association between CD2BP2‐DT expression and clinicopathological parameters in breast cancer patients was evaluated using Fisher's exact test or chi‐square test. All statistical analyses were conducted using SPSS version 19.0 (IBM, Armonk, NY, USA) and GraphPad Prism version 8.0 (La Jolla, CA, USA).

## Conflict of Interest

The authors declare no conflict of interest.

## Author Contributions

D.Z., T.J., and H.W. contributed to the conception and design of the study. H.W., B.Z. prepared the figures and drafted the manuscript. D.Z. and T.J. reviewed and revised the manuscript. H.W., B.Z., and J.Z. performed the in vitro and in vivo experiments. H.W. and B.Z. conducted the bioinformatics analysis. Q.H., L.Z., Y.Z., Y.C., and Y.Q. participated in the collection, follow‐up, detection, and data analysis of clinical specimens. All authors read and approved the final manuscript.

## Supporting information



Supporting Information

## Data Availability

The data that support the findings of this study are available from the corresponding author upon reasonable request.
