# Supplementary material for: N4‐Acetylcytidine‐Mediated CD2BP2‐DT Drives YBX1 Phase Separation to Stabilize CDK1 and Promote Breast Cancer Progression
Source: Adv Sci (Weinh). 2025 Feb 20;12(15):2411834. doi: 10.1002/advs.202411834 (PMC12005790; doi:10.1002/advs.202411834)
Supplement: Supplementary file 1 — Supporting Information [file ADVS-12-2411834-s001.docx]

**N4-acetylcytidine-mediated CD2BP2-DT Drives YBX1 Phase Separation to Stabilize CDK1 and Promote Breast Cancer Progression**

**Authors:**

*Hongyu Wang,* *Bozhi Zhao, Jiayu Zhang, Qunyu Hu, Linlin Zhou, Yinghui Zhang,* *Yixin Cai, Yuansong Qu, Tao Jiang*,* *Dongwei Zhang**

**Supporting Information**

**Figure S1.**

**
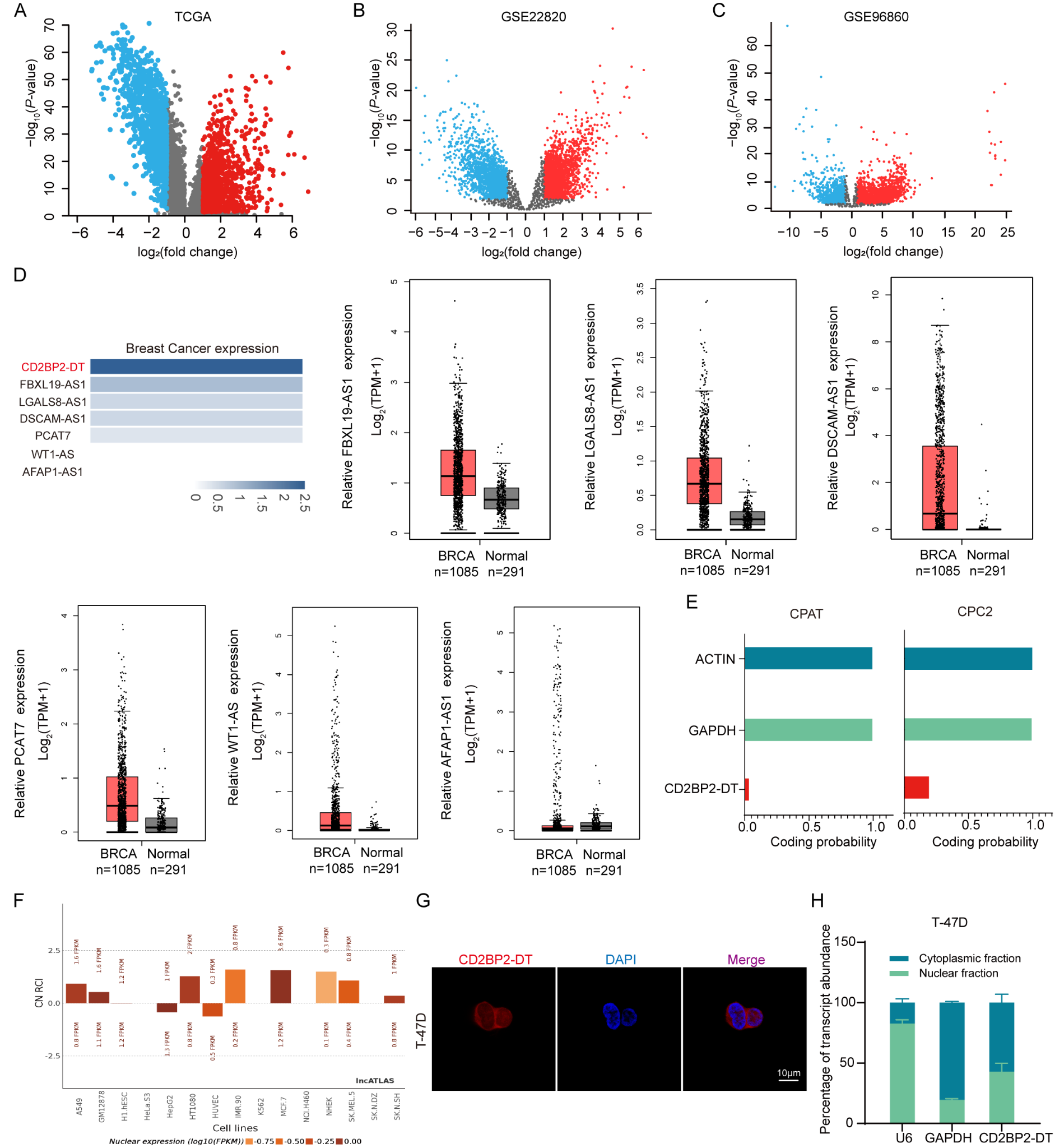
**

**Figure S1.** Identification of CD2BP2-DT and analysis of its expression in breast cancer. A) Volcano plot of DEGs in breast cancer tissues and normal samples in the TCGA database. B) Volcano plot of DEGs in breast cancer tissues and normal samples in the GSE22820 dataset. C) Volcano plot of DEGs between breast cancer cells and normal mammary epithelial cells in the GSE96860 dataset. D) Expression of lncRNAs in the TCGA and GTEx databases (BRCA, n = 1085; Normal, n = 291). E) Coding potentials of CD2BP2-DT and mRNA (GAPDH, ACTB) were calculated using CPAT and CPC2. F) The subcellular localization of CD2BP2-DT in LncATLAS database. G) Representative RNA-FISH images showing the localization of CD2BP2-DT in breast cancer cells. CD2BP2-DT probes are marked in red, and nuclei are stained with DAPI (scale bars, 10 μm). H) Cytoplasmic and nuclear fractions were extracted from breast cancer cells and analyzed for CD2BP2-DT expression using qRT-PCR. U6 served as the nuclear marker, while GAPDH was used as the cytoplasmic marker. Results are presented as mean ± S.D.

**Figure S2.**

**
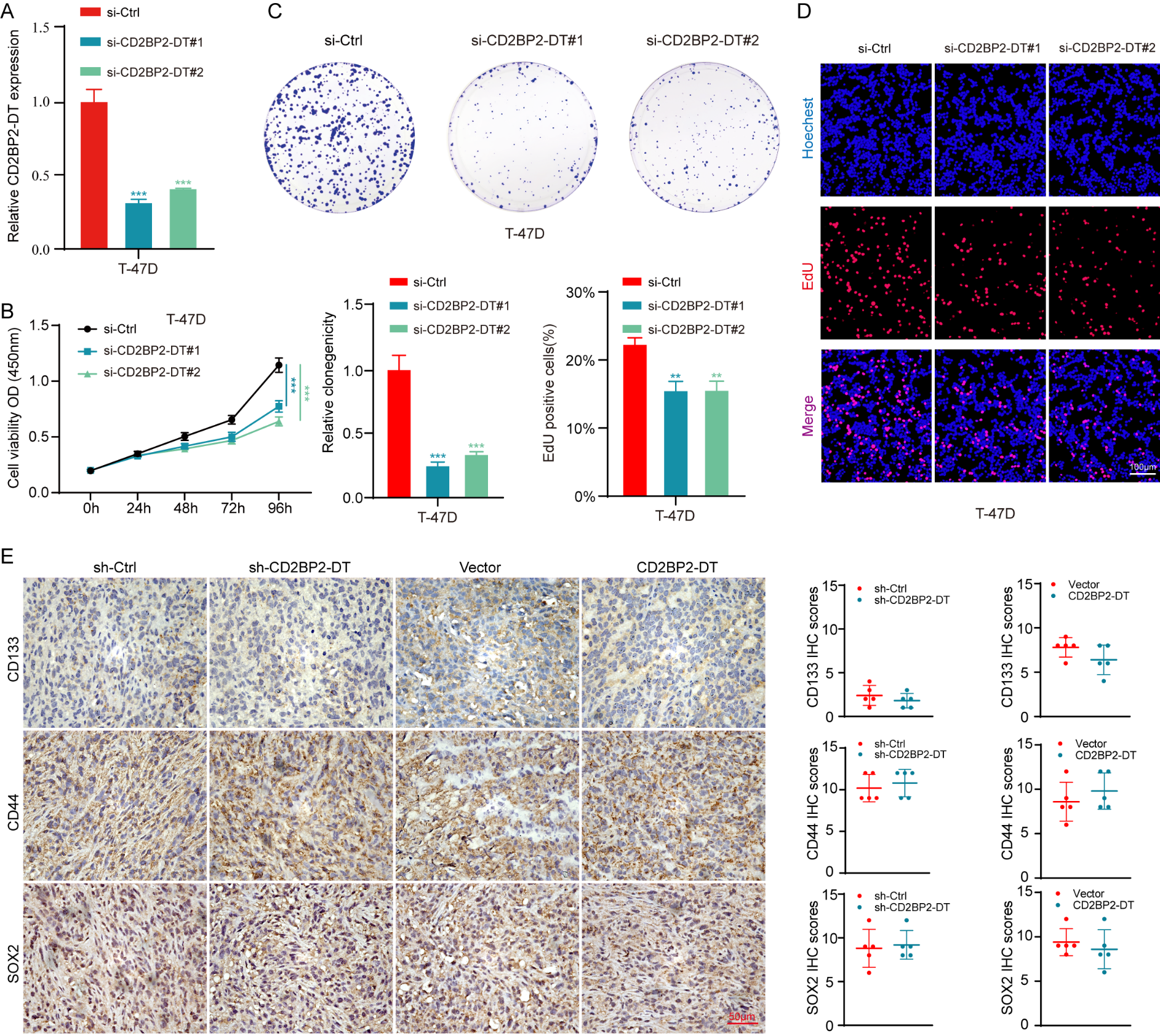
**

**Figure S2.** CD2BP2-DT promotes the growth of breast cancer cells in vitro. A) Assessment of the knockdown efficiency of CD2BP2-DT siRNA using qRT-PCR (n = 3). B) The viability of breast cancer cells with CD2BP2-DT knockdown was assessed using CCK-8 assays at the specified time points (n = 3). C, D) Colony formation (C) assays and (D) EdU assays were performed to assess the proliferation ability of breast cancer cells with CD2BP2-DT knockdown (n = 3; scale bars, 100 μm). E) Representative images of IHC staining for CD133, CD44, and SOX2, in xenograft tumor tissues from different experimental groups (n = 5; scale bars, 50 μm). Data in (A), (C), and (D) were analyzed using one-way ANOVA test. Data in (B) were calculated by two-way ANOVA test. Data in (E) were calculated by unpaired Student’s t-test. Results are presented as mean ± S.D. Significance levels are indicated as ***P* < 0.01, and ****P* < 0.001.

**Figure S3.**

**
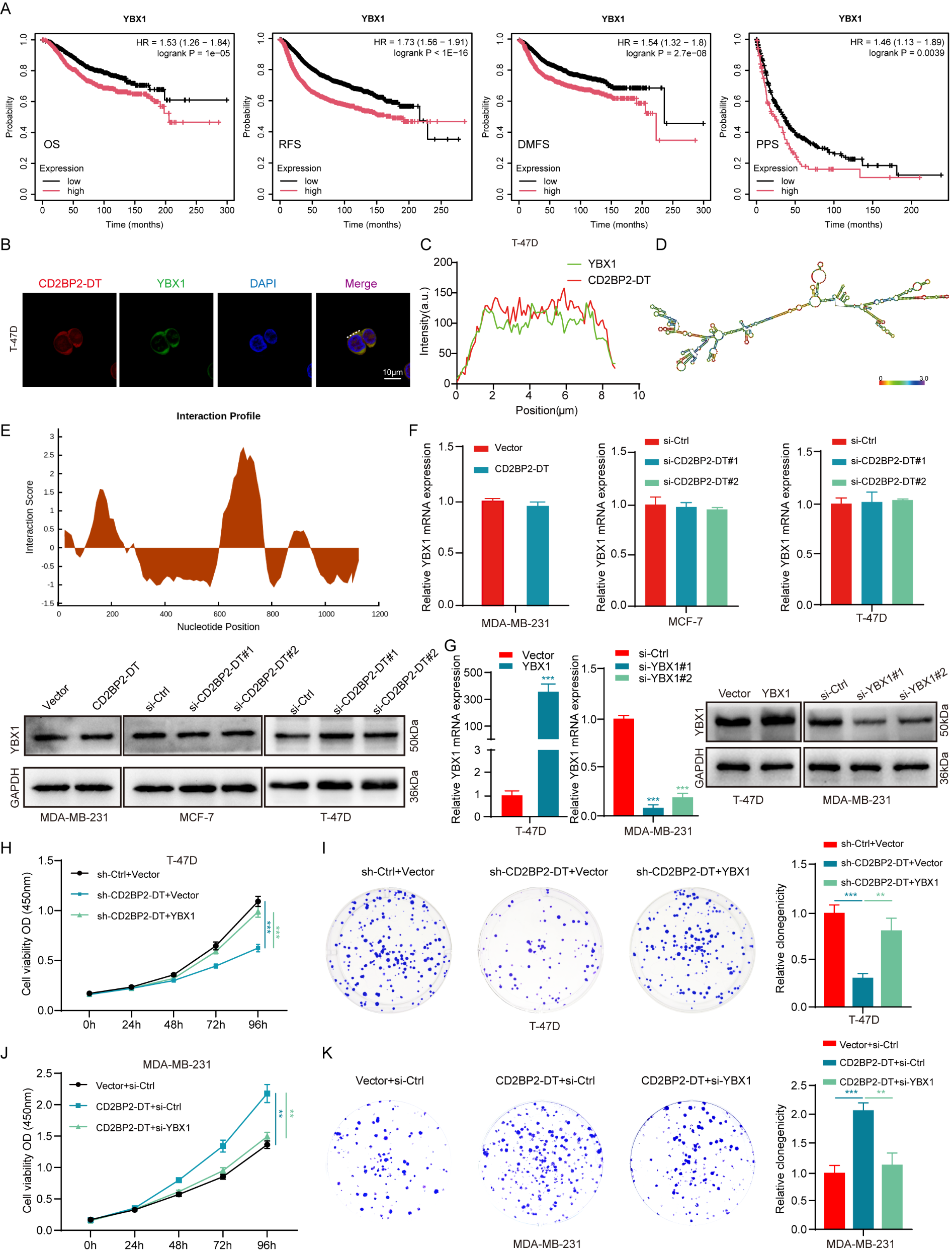
**

**Figure S3.** CD2BP2-DT interacts with YBX1 to promote breast cancer cell proliferation. A) K-M analyses of the correlation between YBX1 mRNA levels and the OS, recurrence-free survival, distant metastasis-free survival, and post-progression survival rates of breast cancer patients, using the Kaplan-Meier Plotter database. B) RNA FISH and immunofluorescence experiments were conducted to analyze the co-localization of CD2BP2-DT with YBX1 (scale bars, 10 μm). C) The fluorescence co-localization was statistically analyzed using image J software. D) Secondary structure of CD2BP2-DT. E) The interaction regions of YBX1 with CD2BP2-DT were predicted using catRAPID database. F) qRT-PCR and Western blotting analysis showing the expression of YBX1 in breast cancer cells transfected with CD2BP2-DT knockdown or overexpression constructs (n = 3). G) qRT-PCR and Western blotting analysis showing the expression of YBX1 in breast cancer cells transfected with YBX1 knockdown or overexpression constructs (n = 3). H, I) CCK-8 (H) and colony formation (I) assays were used to analyze the effects of YBX1 overexpression on CD2BP2-DT-mediated breast cancer cell proliferation (n = 3). J, K) CCK-8 (J) and colony formation (K) assays were used to analyze the effects of YBX1 knockdown on CD2BP2-DT-mediated breast cancer cell proliferation (n = 3). Data in (A) using the Log-rank test. Data in (F) and (G) were calculated by one-way ANOVA or unpaired Student’s t-test. Data in (H) and (J) were calculated by two-way ANOVA test. Data in (I) and (K) were calculated by one-way ANOVA test. Results are presented as mean ± S.D. Significance levels are indicated as ***P* < 0.01, and ****P* < 0.001.

**Figure S4.**

**
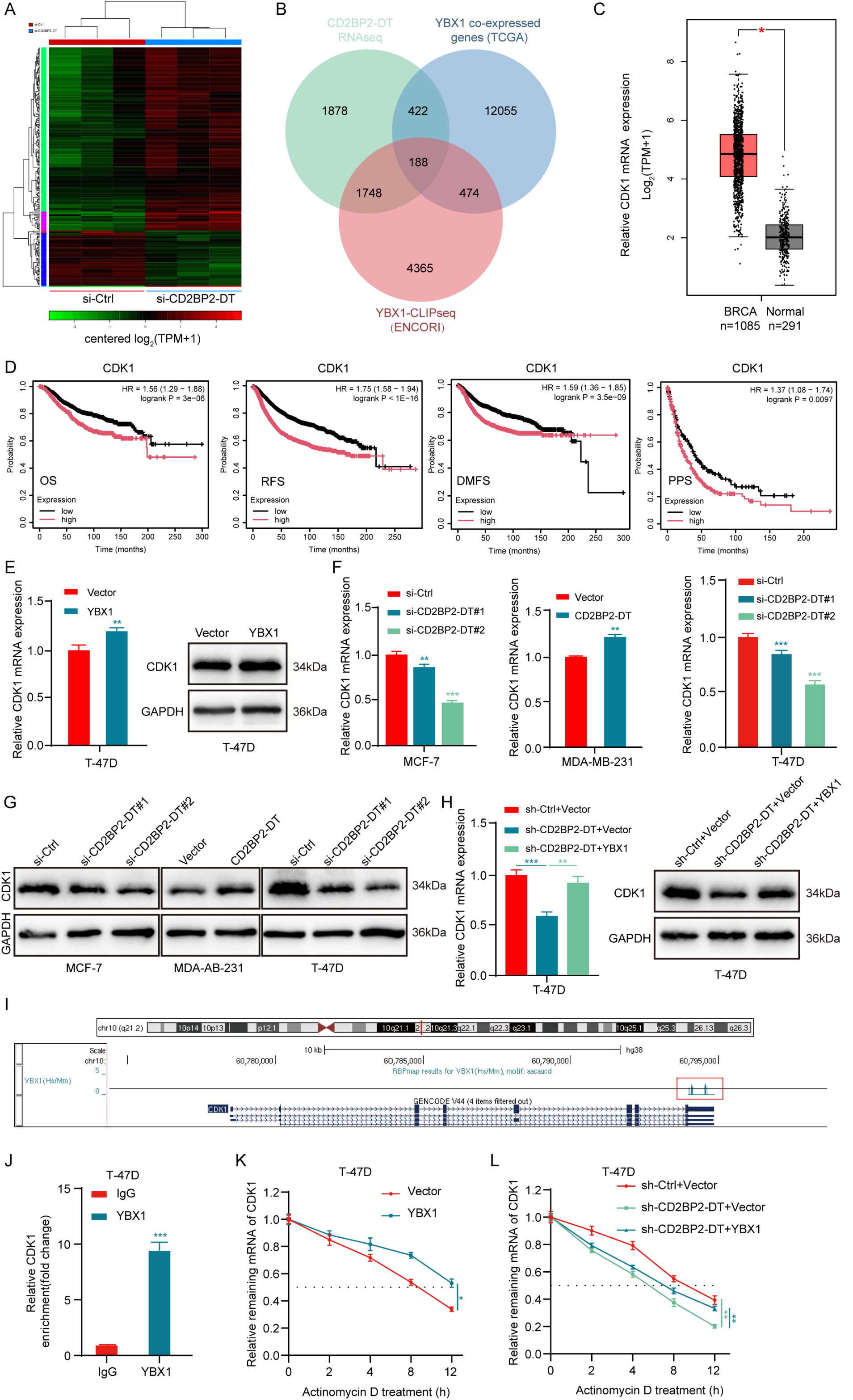
**

**Figure S4.** CD2BP2-DT facilitates the interaction between YBX1 and CDK1 mRNA. A) Heatmaps showing differentially expressed genes after CD2BP2-DT knockdown in breast cancer cells. B) Venn diagram showing the downstream genes co-regulated by CD2BP2-DT and YBX1, along with an overlap analysis with genes potentially bound by YBX1 in CLIP-seq data. C) The CDK1 expression between breast cancer tissues and normal samples in GEPIA 2 database. D) K-M analyses of the correlation between CDK1 mRNA levels and the OS, recurrence-free survival, distant metastasis-free survival, and post-progression survival rates of breast cancer patients, using the Kaplan-Meier Plotter database. E) qRT-PCR and Western blotting analysis showing the expression of CDK1 in breast cancer cells transfected with YBX1 overexpression constructs (n = 3). F, G) qRT-PCR (F) and Western blotting (G) analysis showing the expression of CDK1 in breast cancer cells transfected with CD2BP2-DT knockdown or overexpression constructs (n = 3). H) qRT-PCR and Western blotting were used to analyze the effects of YBX1 overexpression on CD2BP2-DT-mediated CDK1 expression levels (n = 3). I) The binding sites of YBX1 and CDK1 were analyzed using the RBPmap database and visualized through the University of California, Santa Cruz (UCSC) Genome Browser. J) RIP assay indicated CDK1 mRNA was enriched by YBX1 in breast cancer cells (n = 3). K) The remaining CDK1 mRNA was detected using qRT-PCR in breast cancer cells with YBX1 up-regulation after ActD treatment (n = 3). L) The ActD assay was used to analyze the effects of YBX1 overexpression on CD2BP2-DT-mediated stability of CDK1 mRNA (n = 3). Data in (D) using the Log-rank test. Data in (E) and (J) were calculated by unpaired Student’s t-test. Data in (F) were calculated by one-way ANOVA or unpaired Student’s t-test. Data in (H) were calculated by one-way ANOVA test. Data in (K) and (L) were calculated by two-way ANOVA test. Results are presented as mean ± S.D. Significance levels are indicated as **P* < 0.05, ***P* < 0.01, and ****P* < 0.001.

**Figure S5.**


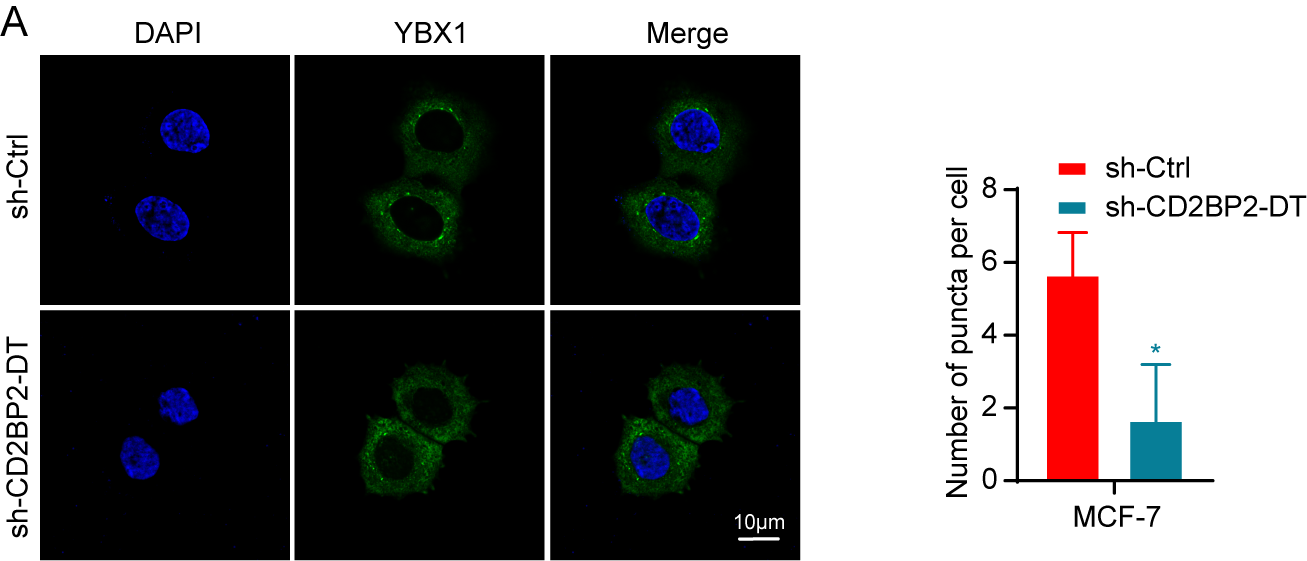


**Figure S5.** CD2BP2-DT induces LLPS of YBX1. A) sh-Ctrl or sh-CD2BP2-DT was transfected with breast cancer cells with YBX1-GFP overexpression, and the breast cancer cells were subsequently analyzed using confocal microscopy and statistically analyzed (n = 3; scale bars, 10 μm). Data in (A) were analyzed using unpaired Student’s t-test. Results are presented as mean ± S.D. Significance levels are indicated as **P* < 0.05.

**Figure S6.**


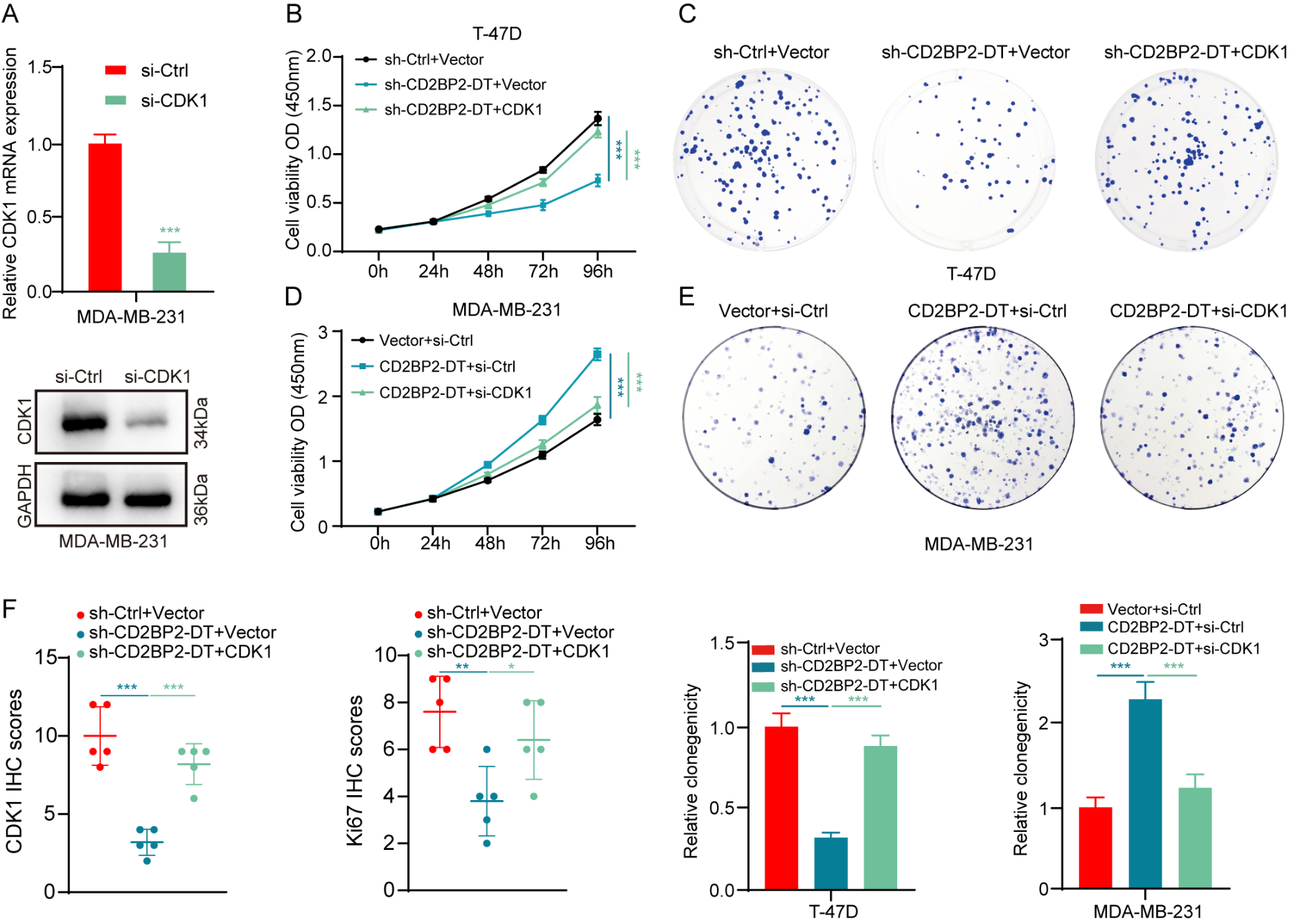


**Figure S6.** CD2BP2-DT/CDK1 axis promotes the proliferation of breast cancer cells. A) The knockdown efficiency of CDK1 was assessed using qRT-PCR and western blot analysis (n = 3). B, C) CCK-8 (B) and colony formation (C) assays were used to analyze the effects of CDK1 overexpression on CD2BP2-DT-mediated breast cancer cell proliferation (n = 3). D, E) CCK-8 (D) and colony formation (E) assays were used to analyze the effects of CDK1 knockdown on CD2BP2-DT-mediated breast cancer cell proliferation (n = 3). F) Assessment of Immunohistochemistry Results Utilizing the IRS Scoring System (n = 5). Data in (A) were calculated by unpaired Student’s t-test. Data in (B) and (D) were calculated by two-way ANOVA test. Data in (C), (E), and (F) were analyzed using one-way ANOVA test. Results are presented as mean ± S.D. Significance levels are indicated as **P* < 0.05, ***P* < 0.01, and ****P* < 0.001.

**Figure S7.**

**
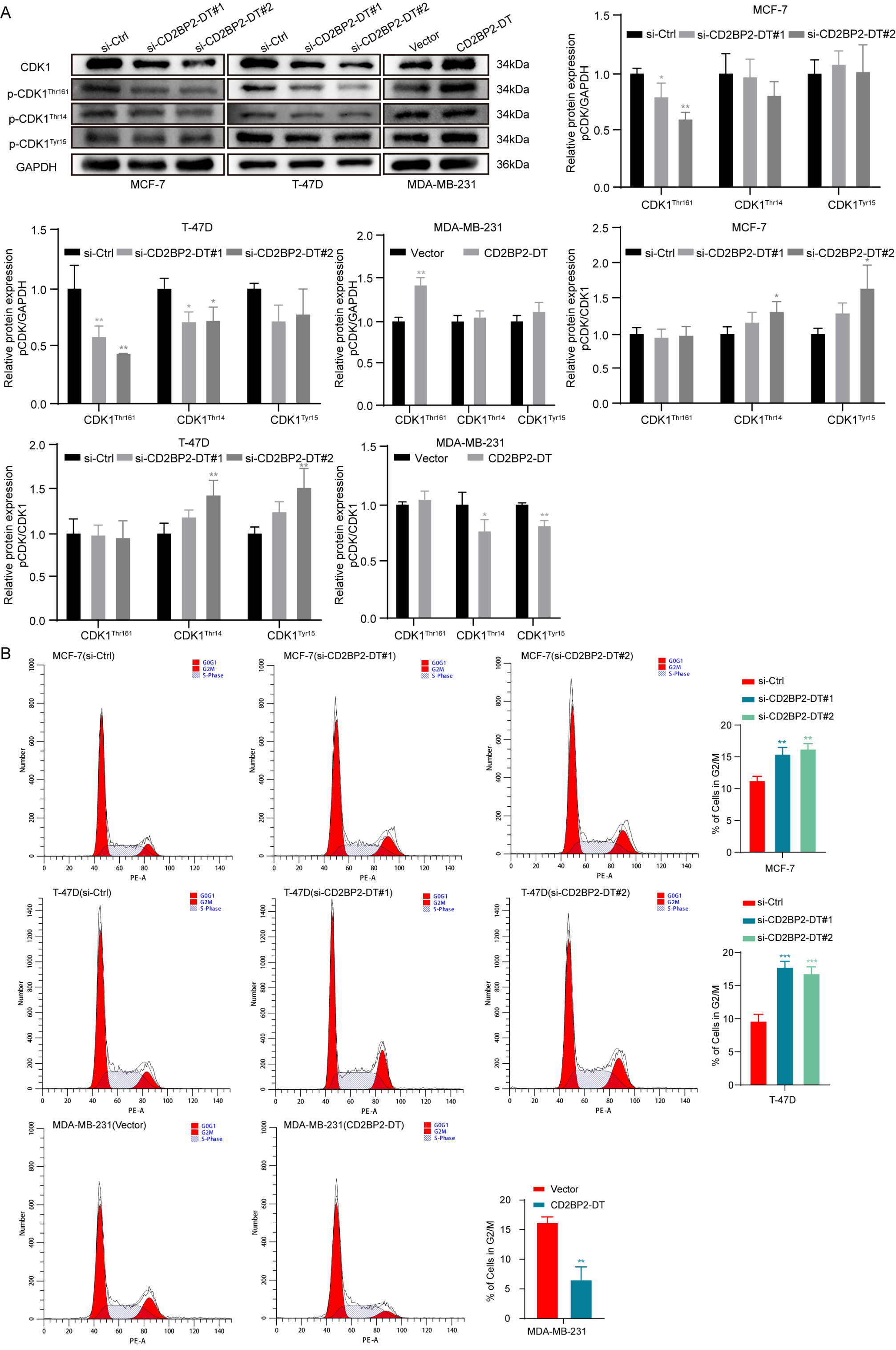
**

**Figure S7.** CD2BP2-DT enhances the expression of CDK1, thereby facilitating the transition from the G2 to M phase in breast cancer cells. A) Statistical Analysis of Protein Blotting and Gray Value Assessment of CDK1 and pCDK1 Alterations Following Knockdown or Overexpression of CD2BP2-DT (n = 3). B) Flow cytometric analysis of alterations in the cell cycle of breast cancer cells following CD2BP2-DT knockdown or overexpression (n = 3). Data in (B) was calculated by one-way ANOVA test or unpaired Student’s t-test. Results are presented as mean ± S.D. Significance levels are indicated as **P* < 0.05, ***P* < 0.01, and ****P* < 0.001.

**Figure S8.**

**
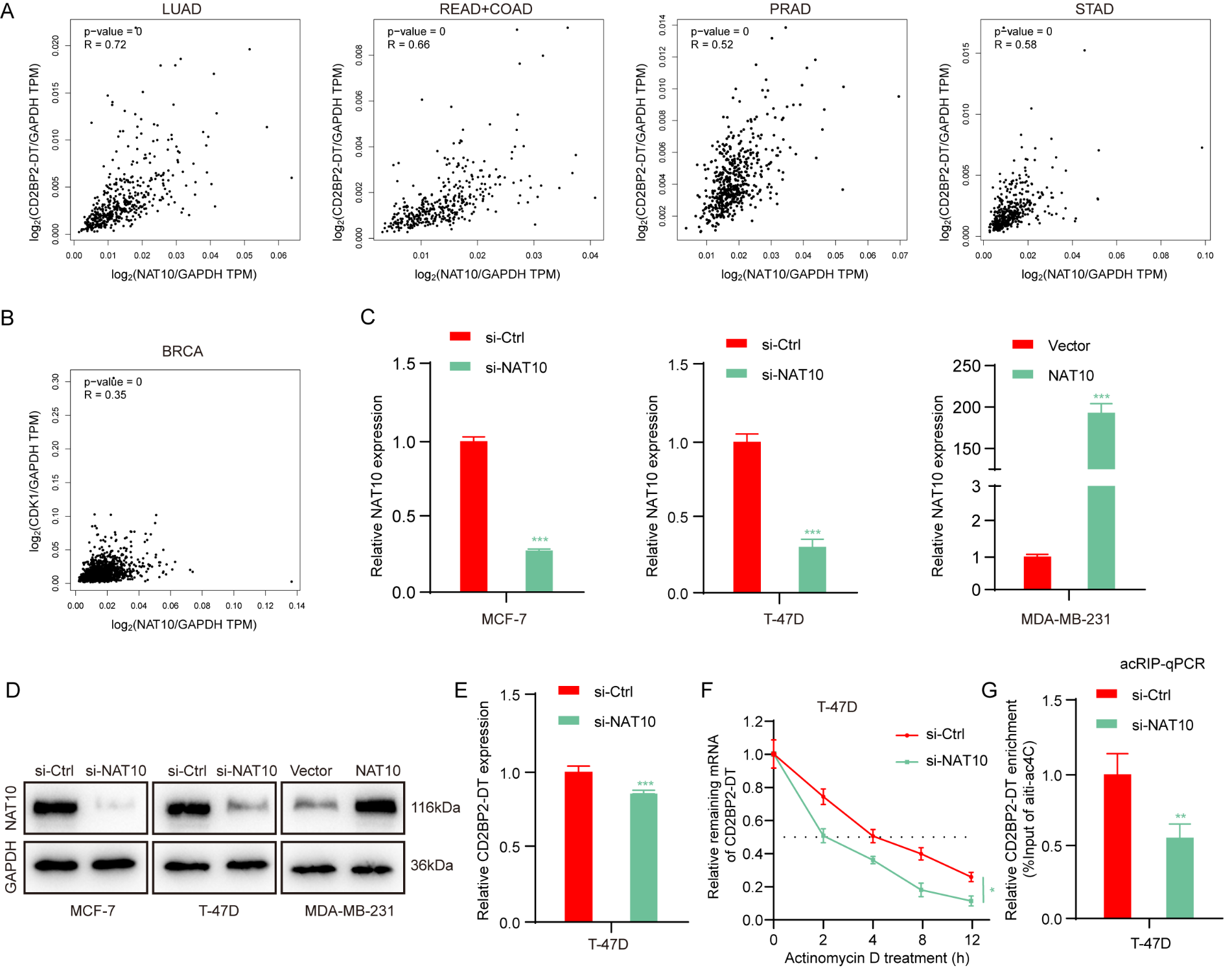
**

**Figure S8.** N4-acetylcytidine modification is involved in the upregulation of CD2BP2-DT in breast cancer. A) The TCGA database was utilized to analyze the correlation between NAT10 mRNA expression and the CD2BP2-DT in cancer. B) The TCGA database was utilized to analyze the correlation between NAT10 mRNA expression and the CDK1 in breast cancer. C) qRT-PCR and D) Western blotting assays were used to determine the efficiency of NAT10 knockdown or overexpression in breast cancer cells (n = 3). E) The expression of CD2BP2-DT was assessed using qRT-PCR following the knockdown of NAT10 (n = 3). F) The degradation rate of CD2BP2-DT was measured in breast cancer cells transfected with si-NAT10 using the ActD method (n = 3). G) The ac4C-RIP assay was conducted to evaluate the level of ac4C modification on CD2BP2-DT in breast cancer cells (n = 3). Data in (A) and (B) were calculated by the Spearman correlation test. Data in (C), (E), and (G) were calculated by unpaired Student’s t-test. Data in (F) were calculated by two-way ANOVA test. Results are presented as mean ± S.D. Significance levels are indicated as **P* < 0.05, ***P* < 0.01, and ****P* < 0.001.

**Table S1.** Relationship between CD2BP2-DT expression and clinicopathological features in breast cancer patients.

| Variables | Cases | CD2BP2-DT expression (n = 114) | | *P* |
| --- | --- | --- | --- | --- |
|  |  | Low (n = 48) | High (n = 66) |  |
| Age (years) |  |  | | 0.127 |
| <60 | 95 | 43 | 52 |  |
| ≥60 | 19 | 5 | 14 |  |
| Tumor size (cm) |  |  | | 0.012 |
| ≤2 | 29 | 18 | 11 |  |
| >2 | 85 | 30 | 55 |  |
| Lymph node metastasis |  |  | | 0.809 |
| No | 65 | 28 | 37 |  |
| Yes | 49 | 20 | 29 |  |
| TNM stage |  |  | | 0.250 |
| I-II | 97 | 43 | 54 |  |
| III-IV | 17 | 5 | 12 |  |
| Grade |  |  | | 0.246 |
| I-II | 78 | 30 | 48 |  |
| III-IV | 36 | 18 | 18 |  |
| Molecular classification |  |  |  | 0.227 |
| Luminal (A + B) | 46 | 15 | 31 |  |
| HRE2+ | 44 | 22 | 22 |  |
| TNBC ^a)^ | 24 | 11 | 13 |  |
| Ki67 |  |  | | 0.117 |
| ≤14% | 23 | 13 | 10 |  |
| >14% | 91 | 35 | 56 |  |

^a)^TNBC stands for triple-negative breast cancer.

**Table S2.** The sequences of oligonucleotides used in this study.

| siRNAs | |
| --- | --- |
| si-Ctrl sense | UUCUCCGAACGUGUCACGUTT |
| si-CD2BP2-DT#1 sense | GCCUCAUCCUGUUAGAGAATT |
| si-CD2BP2-DT#2 sense | GAUGGACCCAAAGCUAAGATT |
| si-YBX1#1 sense  si-YBX1#2 sense  si-CDK1 sense | CCACGCAAUUACCAGCAAATT  GACGGCAAUGAAGAAGAUAATT  GGUUAUAUCUCAUCUUUGATT |
| si-NAT10 sense | CCGAAUCCGGAUUCUCAUUTT |
| shRNAs |  |
| sh-Ctrl sense | TTCTCCGAACGTGTCACGT |
| sh-CD2BP2-DT sense | GATGGACCCAAAGCTAAGA |

**Table S3.** The sequences of primers used for qRT-PCR.

| Primer names | Primer sequences |
| --- | --- |
| CD2BP2-DT | Forward: CTGCTAAAGAAGGGGACCCG |
|  | Reverse: GGGGTCAGGATGGTAAAGGC |
| CDK1 | Forward: AAACTACAGGTCAAGTGGTAGCC |
|  | Reverse: TCCTGCATAAGCACATCCTGA |
| YBX1 | Forward: TAGACGCTATCCACGTCGTAG |
|  | Reverse: ATCCCTCGTTCTTTTCCCCAC |
| NAT10 | Forward: ATAGCAGCCACAAACATTCGC |
|  | Reverse: ACACACATGCCGAAGGTATTG |
| U6 | Forward: CTCGCTTCGGCAGCACA |
|  | Reverse: AACGCTTCACGAATTTGCGT |
| GAPDH | Forward: CTGGGCTACACTGAGCACC |
|  | Reverse: AAGTGGTCGTTGAGGGCAATG |
